# Targeted mutagenesis in a human-parasitic nematode

**DOI:** 10.1371/journal.ppat.1006675

**Published:** 2017-10-10

**Authors:** Spencer S. Gang, Michelle L. Castelletto, Astra S. Bryant, Emily Yang, Nicholas Mancuso, Jacqueline B. Lopez, Matteo Pellegrini, Elissa A. Hallem

**Affiliations:** 1 Molecular Biology Institute, University of California, Los Angeles, California, United States of America; 2 Department of Microbiology, Immunology, and Molecular Genetics, University of California, Los Angeles, California, United States of America; 3 Department of Pathology and Laboratory Medicine, David Geffen School of Medicine, University of California, Los Angeles, California, United States of America; 4 Department of Molecular, Cell and Developmental Biology, University of California, Los Angeles, California, United States of America; George Washington University School of Medicine and Health Sciences, UNITED STATES

## Abstract

Parasitic nematodes infect over 1 billion people worldwide and cause some of the most common neglected tropical diseases. Despite their prevalence, our understanding of the biology of parasitic nematodes has been limited by the lack of tools for genetic intervention. In particular, it has not yet been possible to generate targeted gene disruptions and mutant phenotypes in any parasitic nematode. Here, we report the development of a method for introducing CRISPR-Cas9-mediated gene disruptions in the human-parasitic threadworm *Strongyloides stercoralis*. We disrupted the *S*. *stercoralis* twitchin gene *unc-22*, resulting in nematodes with severe motility defects. *Ss-unc-22* mutations were resolved by homology-directed repair when a repair template was provided. Omission of a repair template resulted in deletions at the target locus. *Ss-unc-22* mutations were heritable; we passed *Ss-unc-22* mutants through a host and successfully recovered mutant progeny. Using a similar approach, we also disrupted the *unc-22* gene of the rat-parasitic nematode *Strongyloides ratti*. Our results demonstrate the applicability of CRISPR-Cas9 to parasitic nematodes, and thereby enable future studies of gene function in these medically relevant but previously genetically intractable parasites.

## Author summary

Parasitic worms are a widespread public health burden, yet very little is known about the cellular and molecular mechanisms that contribute to their parasitic lifestyle. One of the major barriers to better understanding these mechanisms is that there are currently no available methods for making targeted gene knockouts in any parasitic worm species. Here, we describe the first mutant phenotype in a parasitic worm resulting from a targeted gene disruption. We applied CRISPR-Cas9-mediated mutagenesis to parasitic worms in the genus *Strongyloides* and developed a method that overcomes many of the challenges that have previously inhibited generating mutant parasitic worms. We characterize heritable mutant phenotypes and outline a toolkit that will be applicable to many other genes with potential roles in parasitism. Importantly, we developed our method for gene knockouts in a human-parasitic worm. By directly investigating the genes and molecular pathways that enable worms to parasitize humans, we may be able to develop novel anthelmintic therapies or other measures for preventing nematode infections.

## Introduction

Human-parasitic nematodes cause an annual disease burden of over 5 million disability adjusted life years (DALYs) [1,2]. Current drugs used to treat nematode infections are inadequate to eliminate this disease burden: reinfection rates are high in endemic areas and resistance to the few available anthelmintic drugs is a growing concern [2]. However, the development of new strategies for combating nematode infections has been severely limited by the lack of a method for gene disruption in parasitic nematodes [3]. While gene knockdowns by RNAi have been achieved in a few species, RNAi shows variable efficacy and has been used successfully for only a few genes [3]. Conversely, chemical mutagenesis screens have been used to generate mutant phenotypes but the causative mutations could not be identified [4]. As a result, the molecular mechanisms that drive development, behavior, and infectivity in parasitic nematodes remain poorly understood.

The clustered, regularly interspaced, short palindromic repeats (CRISPR) and CRISPR-associated nuclease Cas9 system [5], which evolved from an immune defense system in bacteria and archaea, has been used for targeted mutagenesis in both model and non-model organisms [6,7]. In this system, Cas9 creates double-strand breaks (DSBs) at the genomic location determined by two small RNAs: a CRISPR RNA (crRNA) complementary to the target site and a *trans*-activating crRNA (tracrRNA). The crRNA and tracrRNA are often synthetically combined into a single guide RNA (sgRNA) [5]. The DSBs are then most commonly repaired through either non-homologous end joining (NHEJ) or homology-directed repair (HDR) pathways, but alternative repair mechanisms have been reported in some cases [8]. Despite its application to a wide range of organisms, the CRISPR-Cas9 system has not been successfully utilized in parasitic nematodes. Reasons for this include the low tolerance of parasitic nematodes for exogenous DNA or protein, the labor-intensiveness and low efficiency of methods for delivering constructs for gene targeting, the need to propagate most parasitic nematodes inside an animal host, and the inaccessibility of host-dwelling life stages to genetic intervention [3].

**Fig 1:**
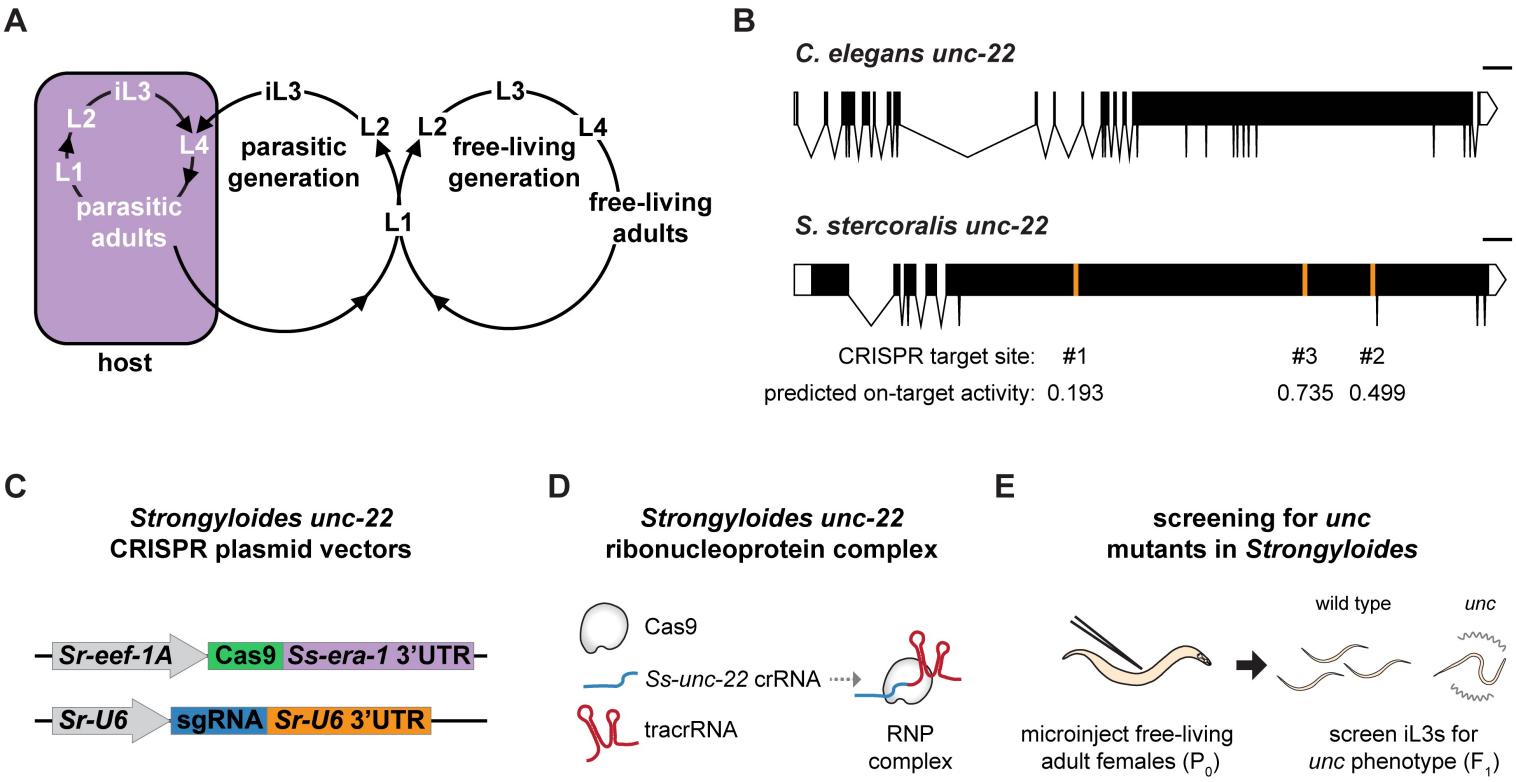
A strategy for targeted mutagenesis in *S*. *stercoralis* using CRISPR-Cas9. (**A**) The life cycle of *S*. *stercoralis*. iL3s enter hosts by skin penetration. The nematodes then develop into parasitic adults, which reside and reproduce in the small intestine. Their progeny exit the host in feces and develop into either iL3s or free-living adults. The free-living adults mate and reproduce in the environment, and all of their progeny develop into iL3s. Thus, *S*. *stercoralis* can develop through a single generation outside the host [10]. *S*. *stercoralis* can also complete its life cycle within a single host [9]. L1-L4 = 1^st^-4^th^ larval stages. Adapted from Gang and Hallem, 2016 [10]. (**B**) The *unc-22* genes of *C*. *elegans* and *S*. *stercoralis*. The *Ss-unc-22* gene structure depicted is based on the gene prediction from WormBase ParaSite [24,47]. The CRISPR target sites tested and their predicted on-target activity scores are indicated [50]. Scale bar = 1 kb. (**C**) Plasmid vectors for the expression of Cas9 and sgRNA in *S*. *stercoralis*. (**D**) RNP complex assembly. Cas9 protein, crRNA targeting *Ss-unc-22*, and tracrRNA are incubated *in vitro* to form RNP complexes [20]. (**E**) Strategy for targeted mutagenesis in *S*. *stercoralis*. Plasmid vectors or RNP complexes were introduced into developing eggs by gonadal microinjection of free-living adult females. F_1_ iL3 progeny were screened for *unc* phenotypes, putatively resulting from mutation of *Ss-unc-22*.

The human-parasitic threadworm *Strongyloides stercoralis* is a powerful model system for mechanistic studies of parasitic nematode biology. *S*. *stercoralis* is a skin-penetrating intestinal nematode that infects approximately 100 million people worldwide; it can cause chronic gastrointestinal distress in healthy individuals but can be fatal for immunosuppressed individuals [9]. *S*. *stercoralis* and closely related species are unique among parasitic nematodes in that they can develop through a single free-living generation outside the host (Fig 1A) [10]. The free-living adults are amenable to transgenesis techniques adapted from the model nematode *Caenorhabditis elegans* [3,11], suggesting they may also be amenable to CRISPR-Cas9-mediated mutagenesis. Preliminary evidence that CRISPR-Cas9 can be used for gene disruptions in *S*. *stercoralis* was reported; however, DNA mutations were detected only at extremely low frequency in pooled populations of worms and individual worms with mutant phenotypes were not observed [11]. Thus, whether CRISPR-Cas9 can be used to study gene function in *S*. *stercoralis* was unclear.

Here we report the use of CRISPR-Cas9 to create loss-of-function DNA mutations and mutant phenotypes in *S*. *stercoralis*. We targeted the *S*. *stercoralis* twitchin gene *unc-22*, and subsequently isolated mutant nematodes with uncoordinated (*unc*) phenotypes characterized by decreased motility, sporadic spontaneous twitching, and persistent twitching when exposed to an acetylcholine receptor agonist. We found that CRISPR-Cas9-induced DSBs at *Ss-unc-22* are resolved by HDR when an appropriate repair template is provided. In the absence of an HDR template we found no evidence for small insertions or deletions (indels) at the target sites tested, but instead observed putative deletions of >500 base pairs at the target locus. We optimized CRISPR-Cas9 targeting conditions for *S*. *stercoralis*, and then demonstrated that *Ss-unc-22* mutations are heritable by passing mutant F_1_ progeny through a host and collecting F_2_ or F_3_ nematodes with *unc* phenotypes. Our results pave the way for mechanistic studies of gene function in parasitic nematodes, which may enable the development of novel targeted therapies to improve human-parasitic nematode control.

## Results

### CRISPR-Cas9 targeting of *Ss-unc-22* causes an uncoordinated phenotype

To assess the functionality of CRISPR-Cas9-mediated mutagenesis in *S*. *stercoralis*, we focused on targeting the *S*. *stercoralis* ortholog of the *C*. *elegans unc-22* gene. *Ce-unc-22* encodes twitchin, a large intracellular muscle protein homologous to mammalian connectin [12,13]. We selected *Ss-unc-22* because *Ce-unc-22* has been successfully mutagenized by multiple methods, including CRISPR-Cas9 [14–17]. In addition, disruption of *Ce-unc-22* results in an easily identifiable *unc* phenotype in both heterozygotes and homozygotes, with mutant nematodes showing dramatically impaired motility and intermittent body twitching [13]. We reasoned that the dominant phenotype resulting from loss of *Ss-unc-22* would enable us to easily identify mutagenized *S*. *stercoralis* in the F_1_ generation even at low mutation frequencies. We introduced *Strongyloides*-specific CRISPR-Cas9 components targeting *Ss-unc-22* into the syncytial gonad of *S*. *stercoralis* free-living adult females (Fig 1A) [11,18]. We identified and tested three CRISPR target sites designed to target Cas9 to the largest exon of *Ss-unc-22* (Fig 1B). CRISPR-Cas9 constructs were delivered into *S*. *stercoralis* adults using two approaches. First, we utilized plasmid vectors to express *Strongyloides-*codon-optimized Cas9 and an sgRNA targeting *Ss-unc-22*. Cas9 was expressed under the control of the promoter for the putative *Strongyloides* elongation factor 1-alpha gene *eef-1A*. *C*. *elegans eef-1A* expresses in the germline, so it was predicted that germline expression would be conserved for *Strongyloides eef-1A* [19]. Expression of the sgRNA was driven by the putative *Strongyloides* U6 promoter (Fig 1C). Second, we targeted *Ss-unc-22* using a ribonucleoprotein (RNP) complex consisting of *in vitro*-assembled recombinant Cas9 protein, crRNA targeting *Ss-unc-22*, and tracrRNA (Fig 1D) [20]. We delivered CRISPR-Cas9 plasmid vectors or RNP complexes into free-living adult females, mated microinjected females with wild-type free-living males, and screened for *unc* phenotypes in F_1_ progeny at the infective third-larval stage (iL3) (Fig 1A and 1E).

**Fig 2:**
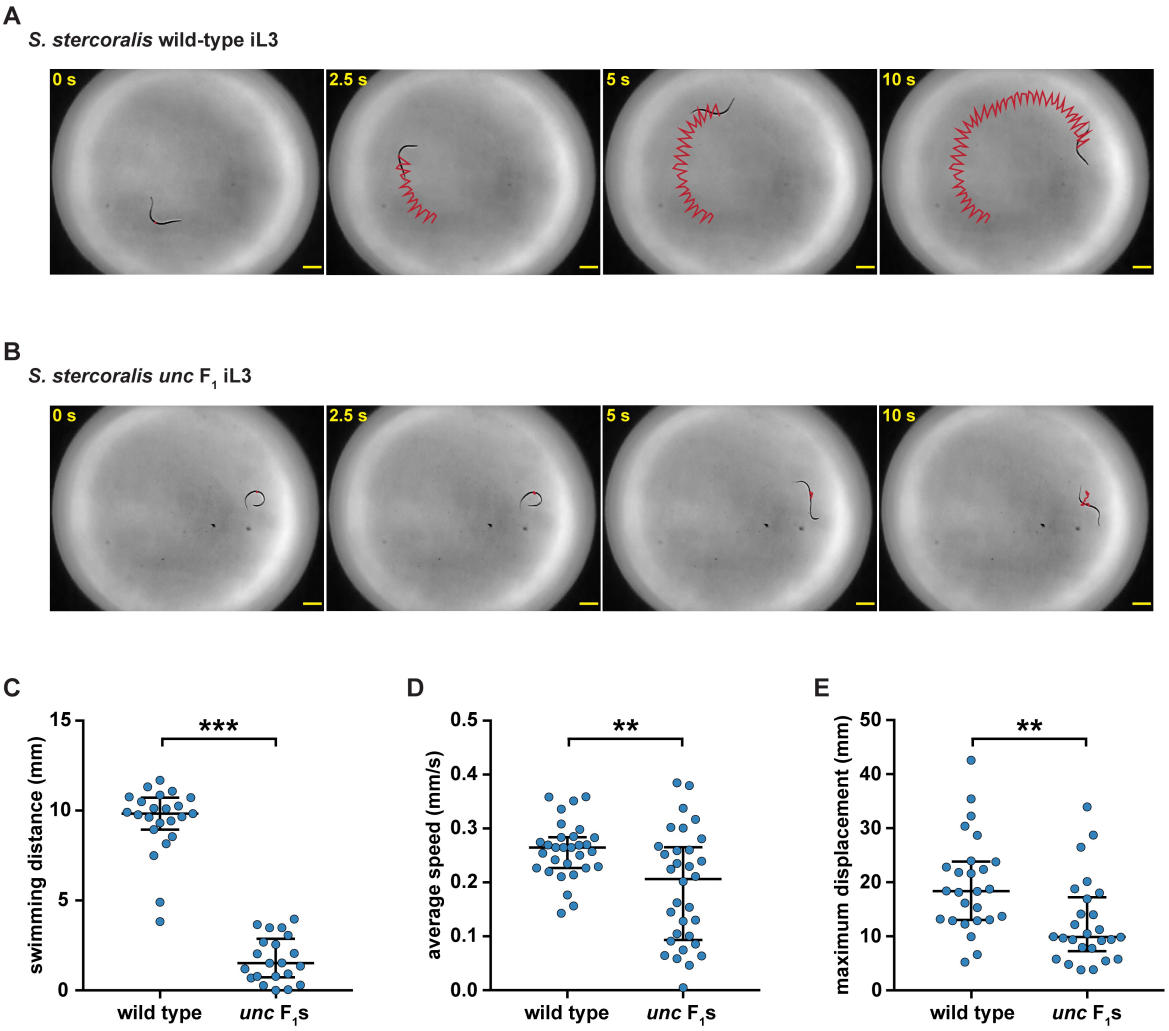
CRISPR-Cas9 targeting of the *Ss-unc-22* gene results in iL3s with an uncoordinated phenotype. (**A-B**) Time-lapse images of wild-type iL3s (**A**) vs. *unc* F_1_ iL3s (**B**) swimming in a water droplet. Wild-type iL3s showed continuous rapid movement in water; *unc* F_1_ iL3s experienced intermittent bouts of twitching, paralysis, and uncoordinated movement. For **A** and **B**, red lines indicate iL3 trajectories. Scale bars = 200 μm. (**C**) Swimming distance for wild-type iL3s vs. *unc* F_1_ iL3s over a 10-s period. *unc* F_1_ iL3s swam shorter distances relative to wild-type iL3s. ***P<0.001, Mann-Whitney test. n = 21–23 trials for each population. (**D**) Average crawling speed for wild-type iL3s vs. *unc* F_1_ iL3s over a 20-s period. *unc* F_1_ iL3s showed reduced crawling speeds relative to wild-type iL3s. **P<0.01, unpaired t test with Welch’s correction. n = 30–32 trials for each population. (**E**) Maximum crawling displacement for wild-type iL3s vs. *unc* F_1_ iL3s over a 5-min period. *unc* F_1_ iL3s traversed less distance than wild-type iL3s. **P<0.01, Mann-Whitney test. n = 26 trials for each population. For **C-E**, graphs show medians and interquartile ranges. *unc* F_1_ iL3 data for **B-E** were obtained from plasmid vector delivery of CRISPR-Cas9 constructs at *Ss-unc-22* site #1.

Following injection of *Ss-unc-22* CRISPR-Cas9 components into free-living adult females, we collected a distinct population of F_1_ iL3s with a striking uncoordinated phenotype (hereafter referred to as *unc* F_1_ iL3s) that was similar to the phenotype observed in *C*. *elegans unc-22* nematodes. *unc* F_1_ iL3s showed impaired swimming behavior when compared to wild-type iL3s collected from non-injected controls (Fig 2A–2C, S1 and S2 Videos). Quantification of iL3 movement using automated tracking software [21] revealed that *unc* F_1_ iL3s showed reduced crawling speeds relative to wild-type iL3s (Fig 2D, S3 and S4 Videos). We then tracked the trajectories of wild-type vs. *unc* F_1_ iL3s over a 5-minute period and found that *unc* F_1_ iL3s traversed significantly less distance than wild-type iL3s (Fig 2E). The swimming and crawling phenotypes of *unc* F_1_ iL3s collected from injections were reminiscent of *C*. *elegans unc* phenotypes and suggested that we had successfully utilized CRISPR-Cas9 to disrupt *Ss-unc-22*.

### The CRISPR-Cas9-induced *unc* phenotype is exacerbated by nicotine exposure

**Fig 3:**
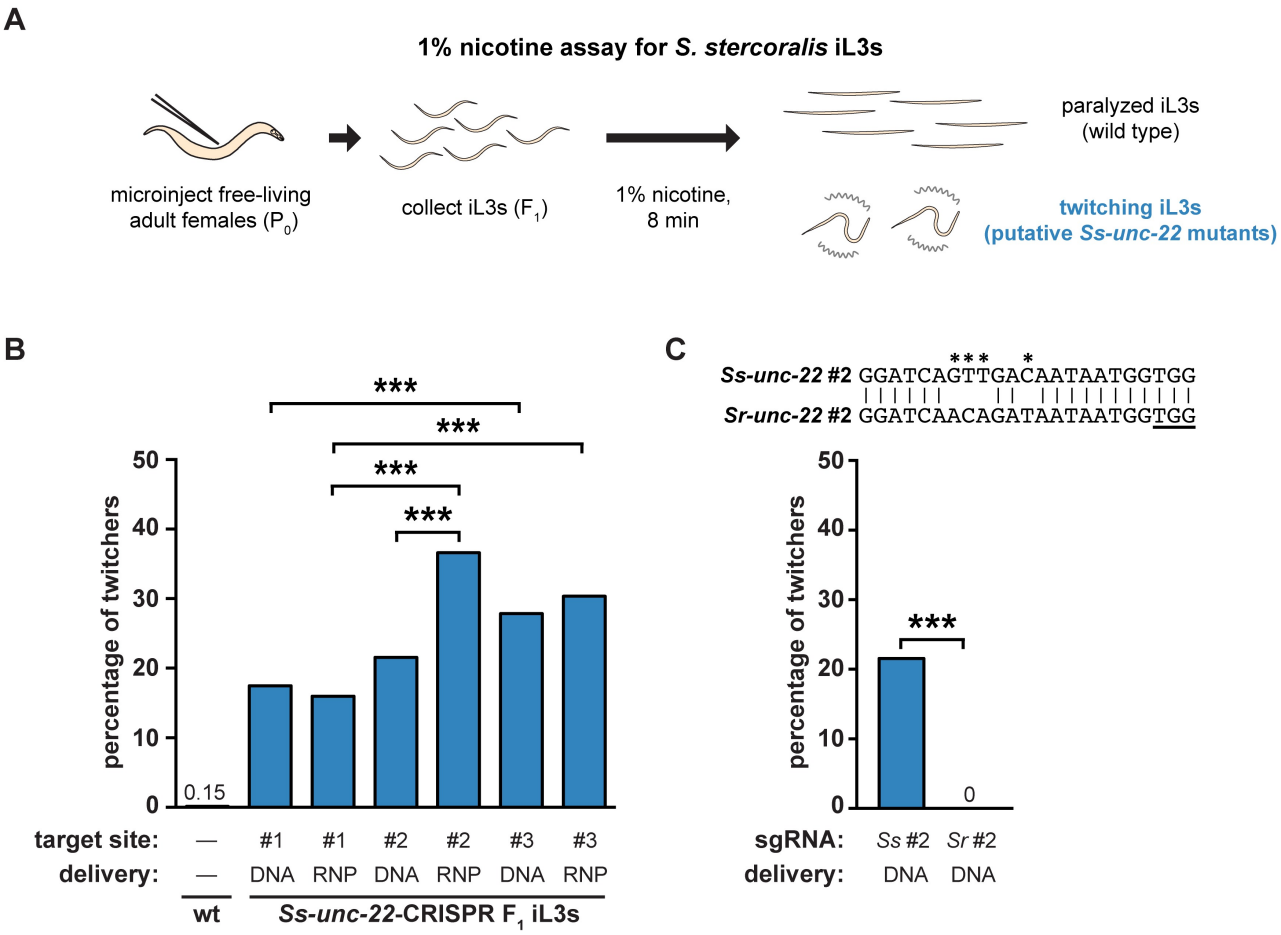
Nicotine induces twitching in *unc* F_1_ iL3s. (**A**) A nicotine assay for *S*. *stercoralis* iL3s. Free-living adult females were injected with CRISPR constructs targeting *Ss-unc-22*. F_1_ iL3s were collected and exposed to 1% nicotine. Wild-type iL3s gradually paralyzed over the course of 8 min, whereas *unc* F_1_ iL3s twitched continuously. Some, but not all, of the F_1_ iL3s contained putative *Ss-unc-22* mutations and twitched in nicotine. (**B**) Twitching frequency of *S*. *stercoralis* wild-type iL3s and the F_1_ iL3s from microinjected females following nicotine exposure. For each condition, the *Ss-unc-22* target site and delivery method of the CRISPR-Cas9 constructs are indicated. DNA = plasmid vector delivery; RNP = ribonucleoprotein complex delivery. The twitching frequency of F_1_ iL3s for all *Ss-unc-22* target sites and delivery methods tested differed from that of wild-type iL3s (*P<*0.001, chi-square test with Bonferroni correction). Instances where twitching frequency differed between target sites or delivery methods are indicated. ***P<0.001, chi-square test with Bonferroni correction. n = 446–1,314 iL3s per condition. (**C**) CRISPR-Cas9-mediated mutagenesis of *Ss-unc-22* requires a highly specific sgRNA. Plasmid vectors for the expression of Cas9 and a sgRNA targeting *S*. *ratti* site #2 were injected into *S*. *stercoralis*. The twitching phenotype in *S*. *stercoralis* F_1_ iL3s was not observed when the *S*. *ratti* version of site #2 was used. ***P<0.001, Fisher’s exact test. n = 484-677 iL3s for each condition. The alignment of *S*. *stercoralis* and *S*. *ratti* site #2 is shown with the PAM underlined. Asterisks indicate nucleotide differences between the *S*. *stercoralis* and *S*. *ratti* targets.

In *C*. *elegans*, the twitching phenotype of *Ce-unc-22* mutants is enhanced by exposure to acetylcholine receptor agonists such as nicotine [13]. We asked if F_1_ iL3s collected following CRISPR-Cas9 injections showed a similar nicotine-induced twitching phenotype. To test this, we developed a nicotine assay for *S*. *stercoralis* iL3s (Fig 3A) and validated it by quantifying twitching behavior in wild-type and *unc-22 C*. *elegans* adults and dauers. We examined dauers as well as adults because the *C*. *elegans* dauer larval stage is a developmentally arrested life stage that is analogous to the parasitic iL3 [22]. Wild-type *C*. *elegans* adults and dauers were completely paralyzed after 8 minutes of nicotine exposure, while *Ce-unc-22* mutants showed severe twitching (S1 Fig). We observed a similar effect in *S*. *stercoralis* iL3s: nicotine induced paralysis in wild-type iL3s but caused nearly continuous twitching in some F_1_ iL3s collected from CRISPR-Cas9 injections (S5 and S6 Videos).

We then used the distinct nicotine-twitching phenotype to assess the efficacy of different *Ss-unc-22* target sites and CRISPR-Cas9 delivery methods. We tested three different CRISPR target sites (Fig 1B) using either plasmid vector or RNP complex delivery of CRISPR-Cas9 components (Fig 1C and 1D). We found that all three target sites, and both CRISPR delivery methods, yielded a population of twitching F_1_ iL3s, and we observed increasing twitching frequency corresponding to increasing predicted on-target activity for each site (Fig 3B, S1 Table). Only site #2 showed a significant difference between plasmid vector and RNP complex delivery, with RNP complex delivery generating a higher frequency of twitching iL3s than plasmid vector delivery (Fig 3B, S1 Table). Overall, we observed a twitching phenotype in ~16–37% of the F_1_ progeny depending on the target-site/delivery-method combination used (S1 Table). Thus, both plasmid vector and RNP delivery methods can be used for CRISPR-Cas9-mediated targeted mutagenesis in *S*. *stercoralis* to similar effect. Importantly, our results show that *unc* F_1_ iL3s (resulting from putative *Ss-unc-22* mutations) can be generated at high efficiency in *S*. *stercoralis* by injecting a moderate number of P_0_ females (S1 Table). Only one free-living generation is accessible for analysis before host infection is required to continue the life cycle. As a result, high efficiency of CRISPR-Cas9 editing in the F_1_ is critical for either immediate investigation of first-generation mutants, or collection of sufficient numbers of mutant progeny to passage through a host and successfully generate a stable mutant line.

We also tested CRISPR-Cas9 activity in *Strongyloides ratti*, a parasite of rats, using the same method outlined for *S*. *stercoralis*. Like *S*. *stercoralis*, *S*. *ratti* can complete a free-living generation outside the host and is amenable to transgenesis [3,11]. We tested two different CRISPR target sites for *Sr-unc-22* using plasmid vector delivery and screened F_1_ iL3s in nicotine (S2A Fig). We found that both target sites yielded a population of twitching F_1_ iL3s, and the nicotine-twitching frequency increased with predicted on-target activity (S2B Fig, S2 Table). The *S*. *ratti* nicotine-twitching phenotype was similar in severity to the phenotype observed in *S*. *stercoralis*. However, the twitching frequency was much lower in *S*. *ratti* than *S*. *stercoralis*, with only ~2–7% of F_1_ progeny displaying the twitching phenotype when injecting a similar number of P_0_ females (S2 Table). Our results demonstrate that CRISPR-Cas9 mutagenesis is applicable in both *S*. *stercoralis* and *S*. *ratti*, two important laboratory models for skin-penetrating parasitic nematode infections [23].

The genomes of *S*. *stercoralis* and *S*. *ratti* are very similar; their *unc-22* genes share >91% sequence identity [24]. The CRISPR target sequences for *Ss-unc-22* site #2 and *Sr-unc-22* site #2 differ by only four base pairs (Fig 3C). We asked whether CRISPR-Cas9 targeting was species-specific by injecting the plasmid vectors encoding Cas9 and the sgRNA for *S*. *ratti* site #2 into *S*. *stercoralis*. We found no evidence for twitching F_1_ iL3s when the *S*. *ratti* version of the sgRNA was used (Fig 3C). Thus, CRISPR-Cas9-mediated mutagenesis of *Ss-unc-22* appears to require highly specific sgRNAs.

### CRISPR-Cas9 mutagenesis causes putative deletion of the *Ss-unc-22* target locus

**Fig 4:**
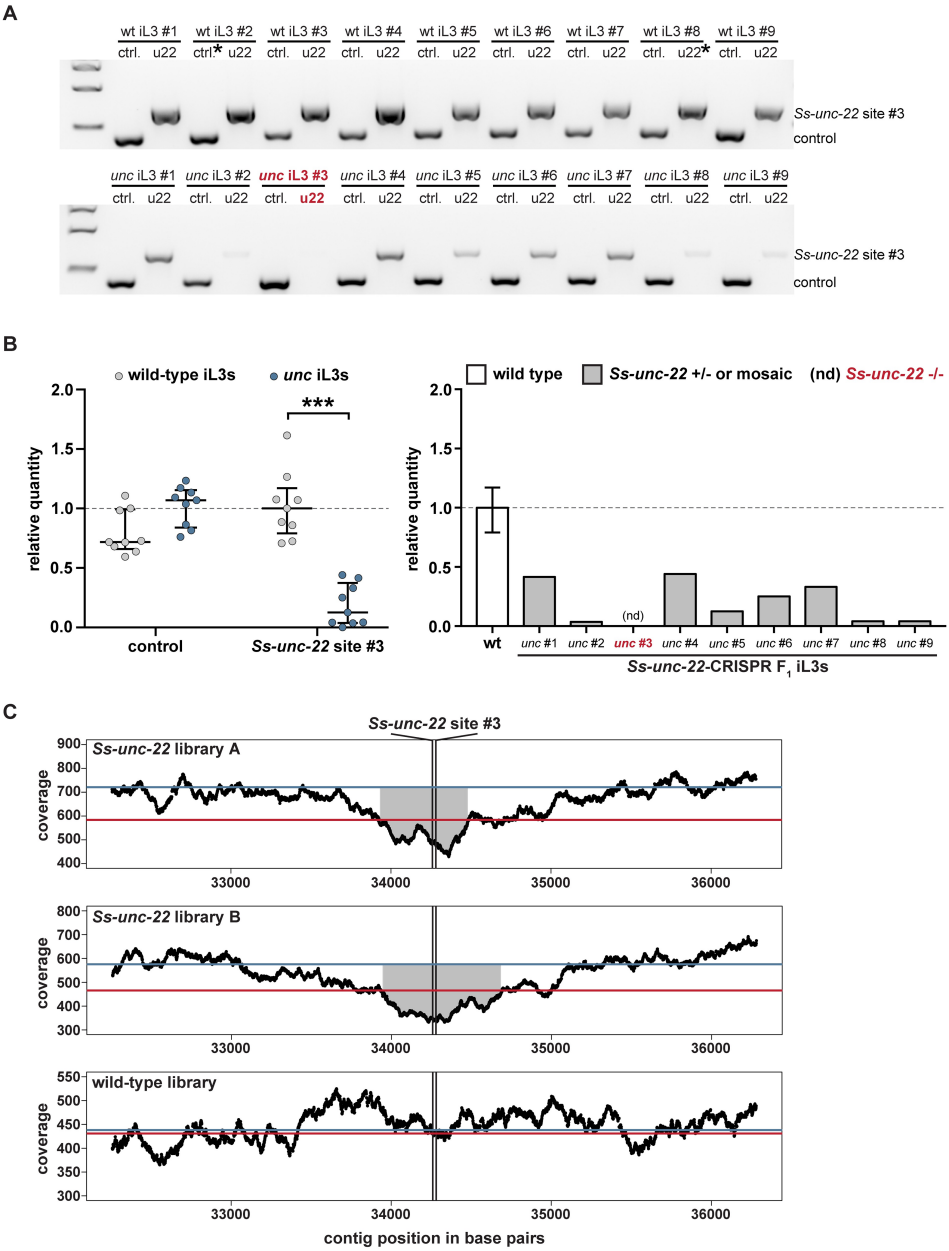
CRISPR-mediated mutagenesis of *Ss-unc-22* results in putative deletion of the target locus. (**A**) Representative gel of wild-type iL3s (top) or *unc* F_1_ iL3s from RNP injections at site #3 (bottom). Genomic DNA from each iL3 was split into two reactions: ctrl. = control reaction amplifying 416 bp of the first exon of the *Ss-act-2* gene to confirm the presence of genomic DNA; u22 = reaction amplifying 660 bp around site #3. Size markers = 1.5 kb, 1 kb, and 500 bp from top to bottom. (**B**) The *Ss-unc-22* region is significantly depleted in *unc* F_1_ iL3s. Left: relative quantity analysis of PCR products. All control bands and all u22 bands were quantified relative to their respective reference bands, denoted by asterisks in **A**. Values >1 indicate more PCR product than the reference while values <1 indicate less product. ***P<0.001, two-way ANOVA with Sidak’s post-test. Medians and interquartile ranges shown. Right: relative quantity of the *Ss-unc-22* site #3 target region for each *unc* F_1_ iL3 tested, and inferred genotypes. nd = PCR product not detected. (**C**) Whole-genome sequencing coverage plots for populations of *Ss-unc-22-*targeted F_1_ iL3s or wild-type iL3s. A 4-kb window centered on the predicted cut site is shown [24,47]. Black lines = average coverage depth by position (reads per base); red lines = average genome-wide coverage; blue lines = average coverage for the *Ss-unc-22* gene. Coverage around *Ss-unc-22* site #3 is significantly depleted in both *Ss-unc-22* libraries relative to the *Ss-unc-22* gene average (*P*<0.05; see Methods). No depletion is observed in the wild-type library (*P>*0.05; see Methods). Gray shaded regions represent stretches of continuous significant coverage depletion around the cut site (*Ss-unc-22* library A = 510 bp, *Ss-unc-22* library B = 725 bp).

Most eukaryotes efficiently repair CRISPR-Cas9-induced DSBs through the NHEJ pathway [5–7,19,25]. NHEJ repair is error-prone and generally introduces small indels near the CRISPR cut site [5]. We asked if the *unc* F_1_ iL3 motility and nicotine-twitching phenotypes observed for *S*. *stercoralis* resulted from CRISPR-Cas9-induced indels at *Ss-unc-22*. For each *Ss-unc-22* target site tested, we collected *unc* F_1_ iL3s that twitched in nicotine (suggesting mutations to *Ss-unc-22*), PCR-amplified the region around the target, and genotyped for indels. Surprisingly, we were unable to detect indels at any of the three *Ss-unc-22* target sites tested. We attempted to identify indels through Sanger sequencing of the target region, heteroduplexed DNA detection by polyacrylamide gel electrophoresis (PAGE) [26], T7E1 endonuclease activity [27], and TIDE (Tracking of Indels by sequence DEcomposition) [28]. For all of the indel detection methods tested, we only observed *Ss-unc-22* wild-type sequence. However, when genotyping individual iL3s, we could reproducibly PCR-amplify the *Ss-unc-22* target region from wild-type iL3s but noted inconsistency in our ability to amplify the *Ss-unc-22* target region from *unc* F_1_ iL3s (Fig 4A). Using control primers, we could however successfully amplify another location in the genome from the same *unc* F_1_ iL3s where the *Ss-unc-22* target region amplified poorly (Fig 4A). Thus, PCR variability was specific to *unc* F_1_ iL3s at the *Ss-unc-22* target region (Fig 4B). Based on the lack of detectable indels in *unc* F_1_ iL3s, we hypothesized that the observed PCR variability at the *Ss-unc-22* target region likely resulted from CRISPR-Cas9-induced deletions that eliminated one, or both, of the primer binding sites. Given that the *C*. *elegans unc* phenotype is dominant [12,13], *unc* F_1_ iL3s where the wild-type band was present are likely heterozygous, or mosaic, deletions of the *Ss-unc-22* target region. *unc* F_1_ iL3s where the band was absent are putative homozygous deletions of *Ss-unc-22* (Fig 4B). We observed putative homozygous deletions for *Ss-unc-22* sites #2 and #3, the more efficient targets, but not *Ss-unc-22* site #1 (S3 Table).

To test the hypothesis that CRISPR-Cas9-mediated mutagenesis results in deletions at the target region, we performed a large-scale microinjection of free-living adults with the RNP complex targeting *Ss-unc-22* site #3. We focused on site #3 since it appeared to produce the most efficient mutagenesis of *Ss-unc-22* (Fig 3B). A single-stranded oligodeoxyribonucleotide (ssODN) was also included in the injection mix to further improve targeting efficiency (described below). We collected a mixed population of wild-type and *unc* F_1_ iL3s, where ~40% of the iL3s displayed the nicotine-twitching phenotype. From this mixed wild-type and *unc* F_1_ population, we prepared two libraries for whole-genome sequencing, as well as a library prepared entirely from wild-type iL3s (S4 Table). We found that the *Ss-unc-22* libraries showed significant depletion in read coverage for an extended stretch of >500 base pairs around *Ss-unc-22* site #3, while no such depletion was observed in the wild-type library (Fig 4C). When targeting *Ss-unc-22* site #3, we found no evidence for read depletion at *Ss-unc-22* site #1 or site #2 in either *Ss-unc-22* library (S3 Fig). Similarly, we found no evidence for read depletion at an unrelated CRISPR target at a distant genomic location in the *Ss-tax-4* gene (see below) (S4 Fig). The observation that read coverage is depleted specifically around site #3 in the *Ss-unc-22* libraries, but not in the wild type, is consistent with the hypothesis that CRISPR-Cas9-mediated mutagenesis results in large deletions rather than small indels at the target locus. To further confirm the lack of small indels, we analyzed indel frequency in the deep-sequencing samples using the CRISPRessoWGS and CRISPRessoCompare computational suite [29]. The CRISPRessoWGS program is designed to analyze deep-sequencing reads aligned to a reference genome and quantify CRISPR-Cas9-editing outcomes, such as indels, at defined targets of interest [29]. We again found no evidence for indels at *Ss-unc-22* site #3 in either *Ss-unc-22* library, suggesting that all of the reads overlapping *Ss-unc-22* site #3 were obtained either from wild-type F_1_ iL3s in the sample or from heterozygous/mosaic *unc* F_1_ iL3s where deletion of the target locus was incomplete.

We note that while >500 base pairs around *Ss-unc-22* site #3 were found to be significantly depleted by whole-genome sequencing, the size of the deletion in many individual *unc* iL3s is likely to be substantially larger. There are a number of possible explanations for why a depleted region of only ~500 base pairs was observed in whole-genome sequencing analysis. First, whole-genome sequencing was performed on a mixed population of iL3s that included both wild-type and *unc* individuals. A mixed population was prepared because, given the labor-intensive nature of the microinjection procedure and screening process for *unc* iL3s, it was impractical to create an all-*unc* population of sufficient density for reliable whole-genome sequencing. As a result, many of the reads from the *Ss-unc-22* libraries were in fact from wild-type individuals. Second, the vast majority of individuals with the *unc* phenotype in the *Ss-unc-22* libraries were mosaics or heterozygotes, and therefore contained wild-type sequence in addition to edited sequence (Fig 4B). Third, each mutant F_1_ iL3 in the *Ss-unc-22* libraries had a potentially different deletion, since the population was not clonal. For these reasons, it is likely that the only region that showed significant depletion is the deleted region that is shared among all of the *unc* iL3s. Many *unc* iL3s may contain larger deletions, as suggested by our PCR results, but these deletions may be positioned asymmetrically around the target site, with the exact breakpoints varying across individuals. The varied nature of these deletions, coupled with the mixture of wild-type and edited DNA sequence, made it impossible to detect larger depletions in the whole-genome sequencing experiments.

Our finding that CRISPR-Cas9-mediated DSBs resulted in deletion of the target locus raised the possibility of unintended disruption of nearby genes. We therefore asked if genomic loci upstream and downstream of *Ss-unc-22* site #3 were intact following CRISPR-Cas9-mediated deletions. To test this, we isolated rare *unc* F_1_ iL3s with putative homozygous deletions of *Ss-unc-22*. For each iL3, we then PCR-amplified regions 10-kb upstream and downstream of the target site; the downstream target amplified in the second exon of the closest gene neighboring *Ss-unc-22* (S5A Fig). For all of the *unc* F_1_ iL3s tested, we successfully amplified the upstream and downstream targets, suggesting that genomic loci near *Ss-unc-22* site #3 are intact following CRISPR-Cas9-mediated deletions (S5B Fig).

We attempted to map the precise endpoints of the *Ss-unc-22* deletion events in *unc* iL3s by PCR-amplifying regions of increasing size, up to 20 kilobases, around the CRISPR target site. However, we never observed PCR products smaller than the wild-type product. We always observed either the wild-type product or no product (Fig 4, S5 Fig). This is likely due to the fact that each deletion in an individual iL3 is potentially unique, making it difficult to optimally design primers to robustly PCR-amplify multiple different deletion events. Further complicating this approach, genomic DNA isolated from individual iL3s is low in concentration, and amplicons of >2–3 kb cannot be amplified reliably. Thus, mapping the endpoints of deletion events was not feasible from individual *unc* iL3s. Additionally, we cannot exclude the possibility that complex chromosomal rearrangements, inversion events, or deletions with inversion events occurred that could not be detected via the methods utilized in this study. Both large deletions and chromosomal rearrangements have been observed in *C*. *elegans* in some cases, and this phenomenon may be more common at certain genomic loci than others [30]. Similar chromosomal rearrangements may have precluded our ability to precisely map CRISPR-mediated mutation events at *Ss-unc-22* targets.

Taken together, our results suggest that CRISPR-Cas9-induced mutations to *Ss-unc-22* are not resolved by small indels near the target, but instead result in deletions around the target site. Importantly, we infer putative homozygous deletions of *Ss-unc-22* in ~2–5% of the *unc* F_1_ iL3s genotyped by PCR (S3 Table). Given the challenges for targeted mutagenesis in parasitic nematodes, the ability to isolate homozygous deletions in the F_1_ generation is critical because it allows for mutant analysis without the need for laborious host passage.

### Homology-directed repair of CRISPR-Cas9-mediated mutations in *S*. *stercoralis*

**Fig 5:**
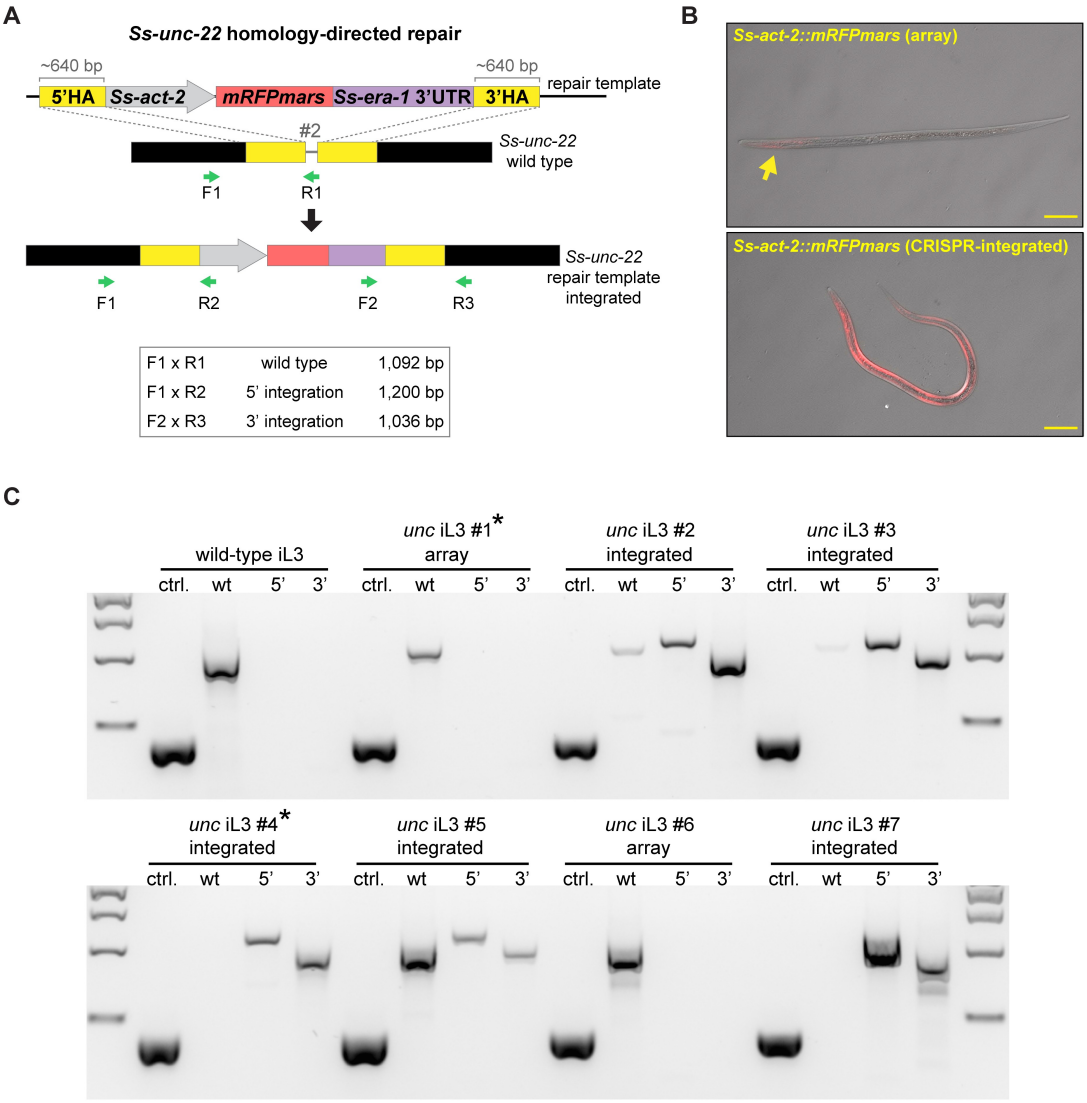
CRISPR-mediated homology-directed repair of *Ss-unc-22*. (**A**) Strategy for HDR at *Ss-unc-22* target site #2. *unc* F_1_ iL3s that displayed both the nicotine-twitching phenotype and red fluorescence were selected as candidates for HDR and were genotyped using the primer sets indicated. 5’ and 3’ integration primer pairs amplify only following successful integration of *Ss-act-2*::*mRFPmars* into site #2. HA = homology arm. (**B**) Representative DIC + epifluorescence overlays of *unc* F_1_ iL3s expressing *Ss-act-2*::*mRFPmars*. Top, iL3 expressing *mRFPmars* (sparse expression indicated by the arrow) from an extrachromosomal array. Bottom, iL3 expressing *mRFPmars* following HDR, showing near-uniform *mRFPmars* expression in the body wall. For both images, anterior is to the left. Scale bar = 50 μm. (**C**) Representative genotypes of a wild-type iL3 and *unc* F_1_ iL3s expressing *mRFPmars*. Genomic DNA from individual iL3s was split into four reactions: ctrl. = control reaction amplifying 416 bp of the first exon of the *Ss-act-2* gene to confirm the presence of genomic DNA; wt = reaction for the wild-type locus of site #2 where primer R1 overlaps the predicted CRISPR cut site; 5’ = reaction for insertion of the 5’ border of the integrated cassette; 3’ = reaction for insertion of the 3’ border of the integrated cassette. For genotypes: array = red *unc* F_1_ iL3s that showed no evidence of integration; integrated = red *unc* F_1_ iL3s with successful HDR. Some integrated iL3s had putative homozygous disruptions of *Ss-unc-22* site #2 (*e*.*g*. iL3s #4 and #7, which lacked the wt band). Asterisks indicate genotypes for iL3s shown in **B**. Size markers = 2 kb, 1.5 kb, 1 kb, and 500 bp from top to bottom.

CRISPR-Cas9-induced DSBs can also be resolved by HDR when a repair template is provided [5]. We asked if DSBs at *Ss-unc-22* could incorporate a repair template containing a fluorescent reporter by HDR, thereby providing an alternative to deletion of the target locus that would facilitate genotyping as well as identification of mutant nematodes. To address this question, we designed a plasmid containing a repair template for *Ss-unc-22* site #2. The repair template consisted of *mRFPmars* under the control of the promoter for the *Strongyloides* actin gene *Ss*-*act-2*, which expresses in contractile filaments of the nematode body wall [31]; the reporter was flanked by homology arms directly adjacent to the CRISPR-Cas9 cut site (Fig 5A). We injected plasmid vectors for the repair template, Cas9, and the sgRNA for site #2 into *S*. *stercoralis*. Interestingly, we found that injections including the repair template increased the percentage of *unc* F_1_ iL3s twitching in nicotine when compared to injections without a repair template (S6A Fig, S5 Table). We then isolated *unc* F_1_ iL3s that displayed a nicotine-twitching phenotype and screened for *mRFPmars* expression, predicting that a subset of these iL3s had successfully repaired CRISPR-Cas9-induced DSBs by HDR. We isolated *unc* F_1_ iL3s with a range of *mRFPmars* expression patterns and fluorescence intensities (Fig 5B). *unc* F_1_ iL3s that expressed *mRFPmars* were genotyped for integration of the repair template using primer sets that spanned the 5’ and 3’ boundaries of the inserted cassette (Fig 5A). We found that >50% of the *unc* F_1_ iL3s that expressed *mRFPmars* showed integration of the repair template at *Ss-unc-22* (Fig 5C, S6 Table). Importantly, we isolated several integrated iL3s that appeared to be putative homozygous knockouts of *Ss-unc-22*: these iL3s were found to lack a wild-type PCR product using a reverse primer that binds the target site (Fig 5C, S6 Table). These results demonstrate the feasibility of generating putative homozygous mutant iL3s in the F_1_ generation using HDR. Sequencing from the 5’ and 3’ boundaries of the repair template confirmed its insertion at the target site (S7 Fig).

In *C*. *elegans*, CRISPR-Cas9-induced DSBs can also be resolved by HDR using an ssODN repair template [20,32]. The ssODN repair strategy has the advantage that ssODNs can be commercially synthesized to allow for rapid CRISPR target testing [20,32]. We asked if ssODNs are also suitable repair templates for HDR in *S*. *stercoralis*. We designed an ssODN for *Ss-unc-22* site #3 (S8A and S8B Fig) and injected it with RNP complexes targeting the same site. Our ssODN was designed in the sense orientation, because sense orientation ssODNs have been shown, in some cases, to be more effective HDR templates in *C*. *elegans* [33]. As with the presence of the *Ss-act-2*::*mRFPmars* repair template, the presence of the ssODN increased the percentage of *unc* F_1_ iL3s twitching in nicotine (S6B Fig, S5 Table). However, we found no evidence for ssODN integration at *Ss-unc-22* site #3 (S8C and S8D Fig). To determine if the absence of ssODN integration was due to the target site selected, or delivery method, we also designed an ssODN for *Ss-unc-22* site #2 and injected it with plasmid vectors. Similarly, we saw no evidence for ssODN integration when using the same target site and delivery method that was used for *Ss-act-2*::*mRFPmars* integration. Our results raise the possibility that incorporation of ssODNs into CRISPR-Cas9-mediated DSBs may not be feasible in *S*. *stercoralis*.

To confirm that integration of the *Ss-act-2*::*mRFPmars* repair template by HDR was specific to CRISPR-Cas9-induced DSBs at *Ss-unc-22*, we repeated injections but removed the Cas9 plasmid vector. We did not observe *unc* F_1_ iL3s twitching in nicotine when Cas9 was omitted, suggesting that *Ss-unc-22* site #2 was not disrupted in the absence of Cas9-induced DSBs (S9A Fig). Similarly, we found no evidence for *unc* F_1_ iL3s twitching in nicotine when Cas9 was omitted from RNP complex injections (S9B Fig). Thus, DSBs at *Ss-unc-22* appear to be specifically triggered by CRISPR-Cas9 mutagenesis.

We next asked if CRISPR-Cas9-mutagenesis coupled with HDR of a fluorescent reporter was applicable to other *S*. *stercoralis* genes. To further validate our HDR approach, we targeted the *S*. *stercoralis* ortholog of the *C*. *elegans tax-4* gene. *Ce-tax-4* encodes a subunit of a cyclic nucleotide gated ion channel that is required for many chemosensory-driven responses in sensory neurons [34,35]. We identified a CRISPR target site for *Ss-tax-4* and modified the *mRFPmars* repair template to contain homology arms near the *Ss-tax-4* CRISPR-Cas9 cut site (S10A and S10B Fig). Following injection of the repair template, Cas9, and the sgRNA for *Ss-tax-4* site #1, we collected F_1_ iL3s and screened for *mRFPmars* expression, again predicting that some of these iL3s would show integration events. As with HDR at *Ss-unc-22*, we isolated *mRFPmars*-expressing F_1_ iL3s that showed *Ss-tax-4* integration events by PCR (S10C Fig, S6 Table). Sequencing from the 5’ boundary of the repair template confirmed its insertion at the *Ss-tax-4* target site (S10D Fig).

Taken together, we conclude that DSBs in *S*. *stercoralis* are specifically triggered by CRISPR-Cas9-mediated mutagenesis, and can be precisely resolved by HDR when a plasmid repair construct is provided. Furthermore, as demonstrated with integration of *Ss-act-2*::*mRFPmars*, repair constructs containing a fluorescent reporter can be used to efficiently screen for gene disruptions in the F_1_ generation. This approach will likely be applicable to many genes of interest in the *S*. *stercoralis* genome.

### Heritable transmission of *Ss-unc-22* mutations

**Fig 6:**
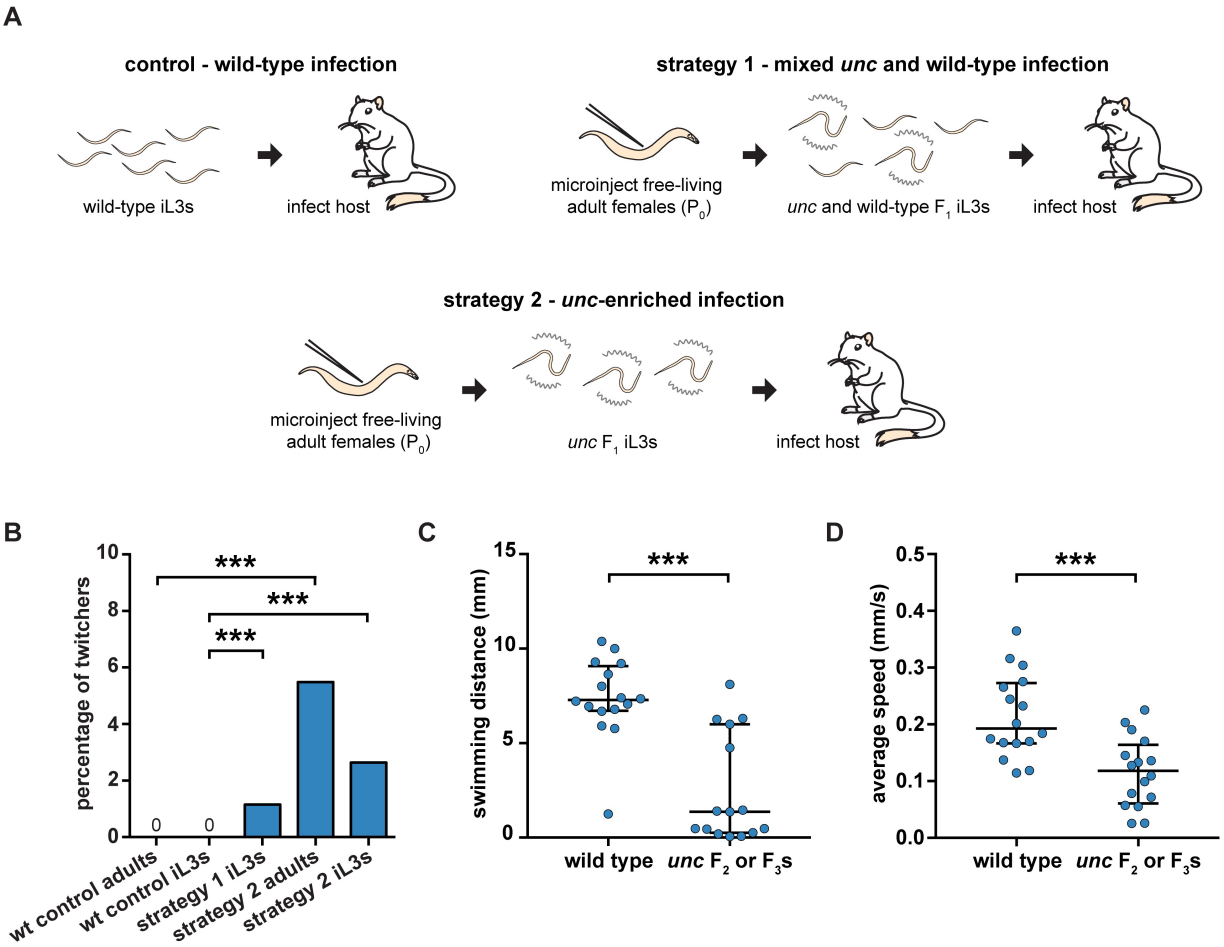
The *unc* phenotype is heritable following host passage. (**A**) Strategies for heritable transmission of *Ss-unc-22* mutations. Gerbil hosts were infected with either all wild-type iL3s, a 50/50 mix of *unc* and wild-type F_1_ iL3s, or *unc*-enriched F_1_ iL3s. F_2_ and F_3_ progeny were collected from host feces and screened for *unc* phenotypes. Note that iL3s collected from host feces can be the F_2_ or F_3_ generation depending on whether they developed into iL3s directly, or after a free-living generation (Fig 1A) [10]. (**B**) Twitching frequency of wild-type control progeny and F_2_ or F_3_ progeny collected from *unc* infections. The twitching frequency of the F_2_ or F_3_ iL3s collected from the mixed *unc* infection differed from that of wild-type iL3s. ***P<0.001, chi-square test with Bonferroni correction. n = 1,908–3,849 iL3s per condition. The twitching frequency of F_2_ adults collected from the *unc*-enriched infection differed from that of wild-type adults. ***P<0.001, chi-square test with Bonferroni correction. n = 164–332 adults per condition. The twitching frequency of F_2_ or F_3_ iL3s collected from the *unc*-enriched infection differed from that of wild-type iL3s. ***P<0.001, chi-square test with Bonferroni correction. n = 2,694–3,849 iL3s per condition. (**C**) Swimming distance for wild-type iL3s vs. *unc* F_2_ or F_3_ iL3s over a 10-s period. *unc* iL3s swam shorter distances than wild-type iL3s. ***P<0.001, Mann-Whitney test. n = 15–16 worms for each population. (**D**) Mean crawling speed for wild-type iL3s vs. *unc* F_2_ or F_3_ iL3s over a 20-s period. *unc* iL3s showed reduced crawling speeds relative to wild-type iL3s. ***P<0.001, unpaired t test with Welch’s correction. n = 16 worms for each population.

One of the challenges for generating targeted gene disruptions in *S*. *stercoralis* is the need to passage F_1_ progeny through a host to maintain the mutation of interest [3]. We developed two strategies to examine if CRISPR-Cas9-induced *Ss-unc-22* mutations are heritable following host passage. First, we injected CRISPR-Cas9 complexes into free-living adult females and collected F_1_ iL3s where approximately 50% of the F_1_ population twitched in nicotine. The mixed population of *unc* F_1_ iL3s and wild-type iL3s was then used to infect gerbils, which are permissive laboratory hosts for *S*. *stercoralis* [36,37]. In a second approach, we injected free-living adult females, collected F_1_ iL3s, enriched for nicotine-twitching *unc* iL3s, and infected gerbil hosts. As a control, we also infected gerbils with exclusively wild-type iL3s (Fig 6A, S7 Table).

Following host infection, we collected host feces from each germline transmission strategy, reared F_2_ and F_3_ progeny, and screened for the nicotine-twitching phenotype as an indicator of successful germline inheritance of *Ss-unc-22* mutations. From the mixed *unc* and wild-type infection strategy, we exclusively screened for the twitching phenotype in F_2_ or F_3_ iL3s. We successfully isolated nicotine-twitching *unc* F_2_ or F_3_ iL3s from the mixed infection over multiple fecal collection days but never observed twitching iL3s from the wild-type control (S7 Video). The average nicotine-twitching frequency of iL3s collected from the mixed infection was 1.2% (Fig 6B, S7 Table). From the *unc*-enriched infection, we screened for the twitching phenotype in both F_2_ free-living adults and F_2_ or F_3_ iL3s. We isolated nicotine-twitching *unc* F_2_ free-living adults from the *unc*-enriched infection but never observed twitching adults from the wild-type control (S8 and S9 Videos). The nicotine-twitching phenotype was observed in ~5% of F_2_ adults (Fig 6B, S7 Table). When we screened F_2_ or F_3_ iL3s from the *unc*-enriched infection, we observed a nicotine-twitching frequency of 2.6% (Fig 6B, S7 Table). An ~5% twitching frequency in F_2_ free-living adults and an ~2.5% twitching frequency in their F_3_ iL3 progeny is consistent with the *unc* phenotype being dominant, and with *unc* F_3_ iL3s resulting from mating events between an *unc* individual and a wild-type individual.

To further validate germline transmission of *Ss-unc-22* mutations, we also characterized the *unc* motility phenotypes of F_2_ and F_3_ iL3s. Twitching F_2_ or F_3_ iL3s were recovered from nicotine and their motility was compared to nicotine-recovered wild-type iL3s. *unc* F_2_ or F_3_ iL3s showed impaired swimming behavior when compared to wild-type iL3s (Fig 6C). Similarly, automated tracking revealed that *unc* F_2_ or F_3_ iL3s showed reduced crawling speeds relative to wild-type iL3s (Fig 6D). The *unc* phenotype observed for F_2_ or F_3_ iL3s was similar to that observed for F_1_ iL3s (Fig 2). Thus, CRISPR-Cas9-mediated mutations are germline-transmissible, and mutant parasites can be propagated by host passage.

## Discussion

Here we demonstrate the first targeted gene disruptions in a parasitic nematode resulting in a mutant phenotype. We exploited the complex life cycle of the human-parasitic threadworm *S*. *stercoralis* to deliver CRISPR-Cas9 constructs targeting *Ss-unc-22* into free-living adults, and characterized *Ss-unc-22* mutations in iL3 progeny (Fig 1). Using this strategy, we generated free-living adults and infective larvae with severe motility defects and altered nicotine sensitivity (Figs 2, 3 and 6). Furthermore, we optimized CRISPR-Cas9 targeting and obtained putative homozygous knockouts in the F_1_ generation, a development that circumvents the necessity for labor-intensive host passage and allows for the immediate interrogation of mutant phenotypes in parasitic worms (Figs 4 and 5). A similar approach is likely to be immediately applicable to other parasitic nematode species, particularly those in the *Strongyloides* and *Parastrongyloides* genera, which are well-suited for gonadal microinjection of CRISPR-Cas9 plasmid vectors or RNP complexes [23,38,39]. Importantly, our results demonstrate that the CRISPR-Cas9 system is functional in parasitic nematodes and represents the first realistic opportunity for systematic gene knockout studies. With additional technical development, this approach may be adaptable to other parasitic nematodes with environmental life stages, such as numerous plant-parasitic and entomopathogenic nematode species.

Both *S*. *ratti* and *S*. *stercoralis* have been proposed as models for targeted mutagenesis in parasitic nematodes [3,11]. *S*. *ratti* has the advantage of requiring fewer iL3s to infect a host; only ~10-20 *S*. *ratti* iL3s are needed to infect a rat, while >1,000 *S*. *stercoralis* iL3s are needed to reliably infect a gerbil. Thus, *S*. *ratti* has been considered the more efficient option for generating stable lines [3,37,40]. In contrast, *S*. *stercoralis* has been shown to be more tolerant of the gonadal microinjection procedure and F_1_ nematodes express transgenes more efficiently [3,11,40]. We found that CRISPR-Cas9 mutagenesis in *Strongyloides* was strikingly similar to reported transgenesis outcomes; *unc-22* mutagenesis was more efficient in *S*. *stercoralis* than *S*. *ratti*, with 17–28% and 2–7% of F_1_ iL3s twitching in nicotine, respectively, when using plasmid vector delivery of CRISPR-Cas9 and injecting a similar number of P_0_ females (Fig 3, S2 Fig, S1 and S2 Tables). The higher mutagenesis efficiency we observed with *S*. *stercoralis* may reflect its increased tolerance of the microinjection procedure. However, future work targeting a number of different genes in *S*. *stercoralis* and *S*. *ratti* will be necessary to determine whether CRISPR-Cas9-mediated mutagenesis is more efficient in *S*. *stercoralis* at all target sites, or only in certain cases. Importantly, the rate of CRISPR-Cas9-mediated mutagenesis in *S*. *stercoralis* was sufficient for germline-transmission and host propagation, as we collected F_2_ adults and F_2_ or F_3_ iL3s with *unc* phenotypes after host passage (Fig 6). However, we note that long-term propagation of the *Ss-unc-22* mutations was not practical given the low efficiency of F_2_ and F_3_ mutant recovery (Fig 6), presumably because the severe motility defects of *Ss-unc-22* iL3s reduced their ability to establish an infection in the host.

Based on the CRISPR-mediated disruption of *Ss-unc-22* and *Sr-unc-22* presented here, future studies pursuing targeted mutagenesis are likely to be feasible in both *S*. *stercoralis* and *S*. *ratti*, with each system having potential advantages. *S*. *stercoralis*, in addition to its direct health relevance as a human parasite, may prove to be a more valuable system for pursuing rapid investigation of homozygous knockouts in the F_1_ generation. Our results show that F_1_ mutagenesis was efficient enough to generate putative homozygous knockouts of *Ss-unc-22* (Figs 4 and 5). Further adding to the potential utility of *S*. *stercoralis*, a recent study demonstrated that heritable transgenesis is also possible by microinjection of plasmid constructs into the testicular syncytium of free-living males [41]. Future CRISPR-Cas9 strategies simultaneously targeting both *S*. *stercoralis* free-living males and females may further improve the incidence of F_1_ homozygous knockouts. In contrast to *S*. *stercoralis*, gene disruptions in *S*. *ratti* are likely to be easier to maintain through successive rounds of host passage due to a more manageable infective dose. Additionally, given the wealth of information for host-parasite interactions between *S*. *ratti* and the rat host, gene knockout studies focused on host immune response, parasite immune manipulation or evasion, and anthelmintic drug administration may be well-suited to this system [42].

Targeted mutagenesis in parasitic nematodes presents a unique challenge in that mutant progeny must be propagated through a host to continue the life cycle. In some cases, knocking out a gene of interest may interfere with the ability of iL3s to infect a host. For example, we recovered only a small percentage of *unc* progeny in the F_2_ and F_3_ generations following host passage (Fig 6, S7 Table). As mentioned above, one hypothesis for the low frequency of *unc* F_2_ and F_3_ progeny is that *unc* iL3s are disadvantaged relative to wild-type iL3s during host infection. Host infection is a multi-step migratory process; iL3s infect by skin penetration, navigate to the circulatory system, penetrate the lungs, and are then thought to be coughed up and swallowed en route to parasitizing the intestinal tract [9]. We observed severe motility defects in *unc* F_1_ iL3s that may handicap their ability to migrate inside the host (Fig 2). Supporting the hypothesis that *unc* motility defects might impede host infection, we consistently recovered fewer F_2_ and F_3_ nematodes from the host feces of *unc-*enriched infections than wild-type infections, despite infecting gerbil hosts with similar numbers of F_1_ iL3s (S7 Table). Mutagenesis studies targeting genes that are essential for parasitic nematodes to infect or develop within the host could be difficult to maintain over multiple generations, although it may be possible to maintain recessive mutations in these genes by passaging heterozygous iL3s through hosts. CRISPR-Cas9 mutagenesis that can efficiently generate homozygous knockouts in a single generation may prove to be the most realistic option for mutant analysis in these cases, and we have demonstrated that this approach is feasible in *S*. *stercoralis*. In contrast, target genes that are not required for infectivity or in-host development may be easier to maintain than the *Ss-unc-22* mutations generated here.

Our results suggest that *S*. *stercoralis* can correct CRISPR-Cas9-induced DSBs by HDR when a plasmid repair template is provided, but not an ssODN (Fig 5, S8 and S10 Figs). In the absence of HDR, we found no evidence for indels at the *Ss-unc-22* target sites tested but instead observed putative large deletions of the target locus (Fig 4). Many eukaryotes predominantly use NHEJ as a DSB repair mechanism [5–7,19,25]. However, whether *S*. *stercoralis* is capable of NHEJ remains unclear. In *C*. *elegans*, DSBs in somatic tissues are repaired by NHEJ, but recent work has demonstrated that germline CRISPR-induced DSBs are repaired by polymerase theta (POLQ)-mediated end joining [43]. Interestingly, CRISPR-Cas9 mutagenesis in *polq-1* deficient *C*. *elegans* routinely results in deletions averaging 10–15 kilobases, including at *Ce-unc-22* targets [43]. In addition, CRISPR-mediated deletions have also been observed in systems that are capable of NHEJ repair. For example, a recent report of CRISPR-Cas9 mutagenesis outcomes in mouse embryonic stem cells suggests that ~20% of edited cells resolve mutations by deletions of 250–9500 base pairs [44]. We hypothesize that *S*. *stercoralis* may favor deletion-based repair of DSBs over generating small indels near the cut site. While we were unable to map the precise endpoints of putative deletions at *Ss-unc-22*, our results suggest that the deletions were greater than 500 base pairs in *unc* F_1_ iL3s, and that the regions 10 kilobases upstream and downstream of the target were unaltered (Fig 4, S5 Fig). We cannot, however, rule out the possibility of more complex DSB repair outcomes such as chromosomal rearrangements, inversion events, or rare indels not detected with the methods used here. Further work will be needed to characterize the DSB repair mechanism in the *S*. *stercoralis* germline in more detail.

In practical terms, HDR using a repair template may be the most straightforward application of CRISPR-Cas9-mediated mutagenesis in *Strongyloides* given that: 1) adding a repair template increases overall targeting efficiency (S6 Fig), 2) HDR is sufficient to generate putative homozygous knockouts in the F_1_ generation (Fig 5, S6 Table), 3) HDR results in precise insertion of the construct of interest at the CRISPR target site instead of generating large deletions (Fig 5, S7 and S10 Figs), 4) incorporation of a fluorescent marker, like *mRFPmars*, simplifies mutant identification and isolation (Fig 5B), and 5) an HDR-based gene disruption strategy is likely to be applicable to many targets in the *S*. *stercoralis* genome (S10 Fig).

In-depth molecular studies in parasitic nematodes have not yet been feasible due to the lack of a toolkit for genetic intervention. We have developed the first practical method for targeted gene disruptions in parasitic nematodes using CRISPR-Cas9. Our results provide a foundation for making these previously intractable parasites more accessible to functional molecular analysis, which may accelerate the development of new strategies to prevent human-parasitic nematode infections.

## Materials and methods

### Ethics statement

Gerbils were used to passage *S*. *stercoralis*. Rats were used to passage *S*. *ratti*. All protocols and procedures used in this study were approved by the UCLA Office of Animal Research Oversight (Protocol No. 2011-060-21A), which adheres to AAALAC standards and the *Guide for the Care and Use of Laboratory Animals*.

### Nematodes and hosts

*C*. *elegans* strains were either N2 Bristol (wild type) or CB66 *Ce-unc-22(e66)* and were obtained from the *Caenorhabditis* Genetics Center. *Strongyloides stercoralis* were the UPD strain and *Strongyloides ratti* were the ED321 strain [22]. Male Mongolian gerbils used to maintain *S*. *stercoralis* were obtained from Charles River Laboratories. Female Sprague-Dawley rats used to maintain *S*. *ratti* were obtained from Envigo Laboratories.

### Maintenance of *S*. *stercoralis*

*S*. *stercoralis* was maintained by serial passage in male Mongolian gerbils as described [36]. *S*. *stercoralis* infective third-stage larvae (iL3s) were collected from fecal-charcoal cultures using a Baermann apparatus [36]. iL3s were cleaned of fecal debris by passage through ~0.5% low-gelling-temperature agarose (Sigma-Aldrich, Cat. # A0701) and washed 5 times in sterile 1x PBS. Isoflurane-anesthetized gerbils were inoculated by subcutaneous injection of ~2,250 iL3s suspended in 200 μL sterile 1x PBS. Feces infested with *S*. *stercoralis* were collected during the patency period of infection, between days 14–45 post-inoculation. Fecal pellets were obtained by placing infected gerbils on wire cage racks overnight with wet cardboard lining the cage bottom; fecal pellets were collected the following morning. Fecal pellets were softened with dH_2_O, crushed, and mixed in a 1:1 ratio with autoclaved charcoal granules (bone char from Ebonex Corp., Cat # EBO.58BC.04). Fecal-charcoal cultures were stored in Petri dishes (10-cm diameter x 20-mm height) lined with dH_2_O-saturated filter paper. To collect free-living *S*. *stercoralis* adults, fecal-charcoal cultures were stored at 20°C for 48 h and adults were isolated using a Baermann apparatus. To collect iL3s, fecal-charcoal cultures were stored at 23°C for at least 5 days and iL3s were isolated using a Baermann apparatus.

### Maintenance of *S*. *ratti*

*S*. *ratti* was maintained by serial passage in female Sprague-Dawley rats as described [45]. *S*. *ratti* iL3s were collected from fecal-charcoal cultures using a Baermann apparatus and washed 5 times in sterile 1x PBS. Rats were inoculated by subcutaneous injection of ~800 iL3s suspended in 300 μL sterile 1x PBS. Feces infested with *S*. *ratti* were collected during the patency period of infection, between days 7–23 post-inoculation. Feces from infected rats were collected and made into fecal-charcoal cultures using the same procedure described above. To collect free-living *S*. *ratti* adults, fecal-charcoal cultures were stored at 20°C for 48 h and adults were isolated using a Baermann apparatus. To collect iL3s, fecal-charcoal cultures were stored at 23°C for at least 5 days and iL3s were isolated using a Baermann apparatus.

### Maintenance of *C*. *elegans*

*C*. *elegans* N2 and CB66 were cultured at room temperature on 6-cm Nematode Growth Media (NGM) plates with *E*. *coli* OP50 bacteria using standard methods [46]. Young adult *C*. *elegans* used in nicotine assays were collected directly from NGM plates containing OP50. Dauer larvae used in nicotine assays were collected in dH_2_O by washing them off of NGM plates where all the OP50 had been consumed. Dauers suspended in dH_2_O were pelleted at 1,000 rpm for 2 min and the supernatant was removed. Pelleted nematodes were then treated with 5 mL of 1% SDS for 15 min at room temperature. After SDS treatment, the nematodes were washed 3x with dH_2_O and transferred to a glass dish. Chemically resistant dauer larvae that survived the SDS treatment were selected and tested in nicotine assays.

### Selection of CRISPR target sites for *Ss-unc-22*, *Sr-unc-22* and *Ss-tax-4*

The *Ss-unc-22* gene was identified based on sequence homology with *C*. *elegans unc-22*. Briefly, the *C*. *elegans* UNC-22 (isoform a) amino acid sequence was used as the query in the TBLASTN search tool to search against the *S*. *stercoralis* genome in WormBase ParaSite (PRJEB528, version WBPS9) [24,47]. The *S*. *stercoralis* gene SSTP_0000031900 was predicted as *Ss-unc-22* based on 55.3% pairwise amino acid identity with *Ce-unc-22*; SSTP_0000031900 was also predicted in WormBase ParaSite as a twitchin and an ortholog of *Ce-unc-22* [24,47]. Reciprocal BLAST of SSTP_0000031900 against the *C*. *elegans* genome predicted *Ce-unc-22* as the best hit. BLAST of SSTP_0000031900 against the *S*. *stercoralis* genome revealed no other obvious *unc-22* orthologs. We searched for CRISPR target sites in *Ss-unc-22* exon 7, the largest exon and the exon with the highest degree of conservation to *Ce-unc-22*. Potential CRISPR target sites were identified with Geneious 9 software using the Find CRISPR Sites plugin [48]. We restricted our CRISPR target sites to those with guanine residues in the 1^st^, 19^th^, and 20^th^ positions in the target sequence (GN(17)GG) and the Cas9 PAM sequence (NGG); these guidelines were established in Farboud *et al*. 2015 for highly efficient guide RNA design in *C*. *elegans* [49]. We selected CRISPR target sites with a range of predicted on-target activity scores based on the algorithm developed in Doench *et al*. 2014, where scores range from 0 to 1, with higher scores representing higher predicted activity (Fig 1B) [50]. We discarded CRISPR target sites with off-target scores under 80% based on the algorithm developed in Hsu *et al*. 2013, where potential targets are rated from 0 to 100%, with higher scores indicating less off-target activity [51]. The same approach was taken to identify *Sr-unc-22* (SRAE_X000227400) in the *S*. *ratti* genome (PRJEB125, version WBPS9 on WormBase ParaSite) [24,47]. *S*. *ratti* CRISPR target sites were selected using the same restrictions outlined for *S*. *stercoralis*. The *Ss-tax-4* gene, SSTP_0000981000, was similarly identified based on sequence homology with *C*. *elegans tax-4*, and was also predicted in WormBase ParaSite as an ortholog of *Ce-tax-4* [24,47]. The *Ss-tax-4* CRISPR target was selected using the same restrictions outlined for *Ss-unc-22*. Gene structure diagrams for *S*. *stercoralis* (Fig 1B and S10A Fig) and *S*. *ratti* (S2A Fig) were generated with Exon-Intron Graphic Maker (Version 4, www.wormweb.org).

### Plasmid vectors for targeted mutagenesis with CRISPR-Cas9

A summary of all plasmid vectors used in this study can be found in S8 Table. pPV540 expressing *Strongyloides-*codon-optimized Cas9 under the control of the *S*. *ratti eef-1A* promoter (previously called *eft-*3 in *C*. *elegans* [19]) was a gift from Dr. James Lok. pPV540 includes the *S*. *stercoralis era-1* 3’UTR; *Strongyloides-*specific regulatory elements are required for successful expression of transgenes in *S*. *stercoralis* and *S*. *ratti* [52]. The sgRNA expression vectors for targeting *Ss-unc-22*, *Sr-unc-22*, and *Ss-tax-4* were synthesized by GENEWIZ in the pUC57-Kan backbone. The *S*. *ratti* U6 promoter and 3’ UTR were identified by sequence homology with *C*. *elegans* U6 [19]; 500 bp and 277 bp regions of the *Sr-*U6 promoter and 3’ UTR, respectively, were included in each sgRNA expression vector. For all sgRNA constructs, a non-base-paired guanine was added to the 5’ end (-1 position on the guide) of each sgRNA to improve RNA polymerase III transcription [49]. Target sequences are provided in S9 Table. The repair construct pEY09 was generated by subcloning approximately 640 bp 5’ and 3’ homology arms flanking *Ss-unc-22* site #2 into the *Strongyloides mRFPmars* expression vector pAJ50 (a gift from Dr. James Lok) [31]. *Ss-unc-22* site #2 was chosen for HDR based on the observation that it was targeted efficiently using plasmid-based delivery of CRISPR-Cas9 constructs (Fig 3B). The repair construct pMLC39 was generated by subcloning approximately 1-kb 5’ and 3’ homology arms flanking *Ss-tax-4* site #1 into pAJ50. Primer sets used to amplify the *Ss-unc-22* site #2 and *Ss-tax-4* site #1 5’ and 3’ homology arms from *S*. *stercoralis* genomic DNA can be found in S13 Table. Injection mixes containing plasmid vectors and concentrations used in this study can be found in S10 Table. Plasmid vector injection mixes were diluted to the desired concentration in ddH_2_O, centrifuged at 14,800 rpm on a bench-top centrifuge through a 0.22 μm tube filter (Costar Spin-X Cat. # 8106) for 15 min, and stored at room temperature prior to use in microinjection experiments. The total DNA concentration injected into free-living *Strongyloides* adult females was limited to a maximum of 100 ng/μL, as described in Junio *et al*. 2008 [31].

### Ribonucleoprotein complexes for targeted mutagenesis with CRISPR-Cas9

RNP complexes were assembled *in vitro* essentially as described [20]. Lyophilized crRNAs targeting *Ss-unc-22* were synthesized commercially (Dharmacon Edit-R Synthetic Modified crRNA, 20 nM) and resuspended in nuclease-free dH_2_O to 4 μg/μL. crRNA sequences are provided in S11 Table. Lyophilized tracrRNA was synthesized commercially (Dharmacon U-002000–20, 20 nM) and resuspended in nuclease-free ddH_2_O to 4 μg/μL. Lyophilized ssODN for *Ss-unc-22* site #3 was synthesized commercially (IDT Ultramer DNA Oligo, 4 nM) and resuspended in nuclease-free ddH_2_O to 500 ng/μL. crRNA, tracrRNA, and ssODN stocks were stored at -20°C until use and kept on ice during RNP complex preparation. RNP injection mixes were made as shown in S12 Table and added to 10 μg of lyophilized recombinant Cas9 protein from *Streptococcus pyogenes* (PNA Bio Inc., Cat. #CP01). The solution was centrifuged for 2 min at 13,000 rpm in a bench-top centrifuge and incubated at 37°C for 15 min to assemble RNP complexes. RNP complex solution was stored on ice prior to use in microinjection experiments.

### Microinjection of *Strongyloides* free-living adults

Gonadal microinjection of plasmid vectors or RNP complexes into the syncytial gonad of *S*. *stercoralis* or *S*. *ratti* free-living adult females was performed as described for *S*. *stercoralis*, *S*. *ratti*, and *C*. *elegans* [18,31,45]. Microinjected females were transferred to 6-cm NGM plates containing OP50 for recovery, and free-living wild-type adult males were added for mating. After a minimum injection recovery time of approximately 30 min, NGM plates were flooded with dH_2_O and free-living males and females were transferred to 6-cm fecal-charcoal plates using non-stick sterile worm-transferring tips (BloomingBio, Cat. # 10020-200-B). Uninfected gerbil feces were collected as described above and used to make fecal-charcoal cultures for *S*. *stercoralis*. Uninfected rat feces were collected as described above and used to make fecal-charcoal cultures for *S*. *ratti*. Host feces were used for post-injection incubation based on the observation that the reproductive output of *Strongyloides* free-living adults is better on feces than with standard *C*. *elegans* culturing methods [53]. Fecal-charcoal cultures were maintained at 23°C. After 5–14 days, F_1_ iL3s were recovered from the fecal-charcoal plates using a Baermann apparatus. The average number of F_1_ iL3s collected from feces per injected female for *S*. *stercoralis* and *S*. *ratti* can be found in S14 Table. iL3s were stored in dH_2_O for 1–2 days at room temperature until behavioral analysis and subsequent genotyping. For each *Strongyloides* CRISPR-Cas9 target site and delivery method tested in this study, we microinjected P_0_ adults and screened F_1_ iL3s in a minimum of two separate experiments per condition.

### Swimming assay

F_1_ iL3s were recovered from fecal-charcoal cultures using a Baermann apparatus and stored in a glass dish in 2–5 mL of dH_2_O. Individual iL3s were then transferred in 2–3 μL of dH_2_O to a 10-cm chemotaxis plate [54]. 10-s recordings of the iL3 swimming in the dH_2_O drop were immediately obtained using an Olympus E-PM1 digital camera attached to a Leica M165 FC microscope. Swimming distances were calculated using the ImageJ Manual Tracking plugin by marking the frame-by-frame position change of the nematode centroid during the 10-s recording and summing the total distance traveled. F_2_ or F_3_ wild-type (paralyzed) or *unc* (twitching) iL3s were recovered from 1% nicotine treatment overnight on chemotaxis plates and tested for swimming behavior the next day.

### Automated tracking of iL3 crawling

Automated tracking was performed as described [22]. Briefly, recordings of iL3 movement were obtained with an Olympus E-PM1 digital camera attached to a Leica S6 D microscope. To quantify movement, 3–5 iL3s were placed in the center of a chemotaxis plate in a 5 μL drop of dH_2_O. Once the drop dried, the iL3s were allowed to acclimate to the plate for 10 min. 20-s recordings were then obtained from each iL3, ensuring that each iL3 was only recorded once. Worm movement was quantified using WormTracker and WormAnalyzer software (Miriam Goodman lab, Stanford University) [21]. The following WormTracker settings were used: minimum single worm area = 20 pixels; maximum size change by worm between successive frames = 250 pixels; shortest valid track = 30 frames; auto-thresholding correction factor = 0.001. F_2_ or F_3_ wild-type (paralyzed) or *unc* (twitching) iL3s were recovered from 1% nicotine treatment overnight on chemotaxis plates and tested for crawling behavior the next day.

### iL3 dispersal assay

Recordings of iL3 movement were obtained with a 5-megapixel CMOS camera (Mightex Systems) equipped with a manual zoom lens (Kowa American Corporation) suspended above a 22-cm x 22-cm chemotaxis plate. To quantify unstimulated movement, individuals iL3s were placed in the center of the chemotaxis plate and allowed to acclimate for 10 min. 5-min recordings were then obtained. Images were captured at 1 Hz using Mightex Camera Demo software (V1.2.0) in trigger mode. Custom Matlab code (MathWorks) and a USB DAQ device (LabJack) were used to generate trigger signals. To quantify maximum dispersal distance, the location of individual iL3s during the recording session was manually tracked using the ImageJ Manual Tracking plugin; maximum distance from initial iL3 location was calculated in Microsoft Excel. Researchers were blinded to twitching phenotype during manual tracking; recording sessions were scored in randomized order.

### Nicotine assay

For adult *C*. *elegans* assays, 5–10 young adults were transferred from NGM plates containing OP50 onto a chemotaxis plate using a worm pick. 20 μL of a 1% nicotine solution diluted in dH_2_O was pipetted onto the nematodes. After 8 min in nicotine, individual nematode phenotypes were scored under a dissecting microscope. We characterized four distinct phenotypes: paralyzed = wild-type nicotine response [13]; partially paralyzed = instances where paralysis was nearly complete but we observed minor movements; unaffected = rare instances where the nematode appeared unaffected by nicotine treatment; and twitching = continuous twitching (the *unc-22* nicotine response [13]). The percentage of twitchers was calculated as: % twitchers = (# twitching nematodes) / (total # of nematodes screened) x 100. *C*. *elegans* dauer assays were performed essentially as described above. dH_2_O drops containing 6–10 SDS-recovered dauers were pipetted onto chemotaxis plates. The drops were allowed to dry and 20 μL of 1% nicotine solution was pipetted onto the dauers. After 8 min, phenotypes were scored and quantified as described above. *Strongyloides* free-living adults and iL3s were recovered from fecal-charcoal cultures using a Baermann apparatus and stored in a glass dish in 2–5 mL of dH_2_O. A chemotaxis plate was subdivided into four sections and ~10 μL of dH_2_O containing nematodes was pipetted into each quadrant (~5–10 free-living adults or 20–50 iL3s per quadrant, 20–40 free-living adults or 80–200 iL3s per chemotaxis plate). The drops were allowed to dry and 40–50 μL of 1% nicotine solution was pipetted onto the worms. After 8 min, phenotypes were scored and quantified as described for *C*. *elegans*. For chi-square analysis, paralyzed, partially paralyzed, and unaffected phenotypes were combined into one “non-twitching nematodes” category and compared to “twitching nematodes.”

### *Strongyloides* genomic DNA preparation

To obtain genomic DNA from *S*. *stercoralis* individual iL3s or small pools of iL3s, 1–15 iL3s were transferred to PCR tubes containing 5-6 μL of nematode lysis buffer (50 mM KCl, 10 mM Tris pH 8, 2.5 mM MgCl_2_, 0.45% Nonidet-P40, 0.45% Tween-20, 0.01% gelatin in ddH_2_O) supplemented with ~0.12 μg/μL Proteinase-K and ~1.7% 2-mercaptoethanol. Tubes were placed at -80°C for at least 20 min, then transferred to a thermocycler for digestion: 65°C (2 h), 95°C (15 min), 10°C (hold). Genomic DNA samples were stored at -20°C until use. For long-term genomic DNA integrity (>1 week), iL3 samples were stored undigested at -80°C; thermocycler digestion was then performed immediately before testing. To obtain genomic DNA from large populations of ~5,000-10,000 *S*. *stercoralis* iL3s, we followed the “Mammalian Tissue Preparation” protocol for the GenElute Mammalian Genomic DNA Miniprep Kit (Sigma-Aldrich, Cat. # G1N10); genomic DNA was eluted in 100 μL of dH_2_O and stored at -20°C until use. To obtain genomic DNA from wild-type iL3 populations or *unc* F_1_ iL3 populations for deep sequencing (S4 Table), we followed the “Isolation of Genomic DNA from Tissues” protocol for the QIAamp UCP DNA Micro Kit (Qiagen, Cat. # 56204); genomic DNA was eluted in 15 μL of nuclease-free ultrapure ddH_2_O and stored at -20°C until library preparation.

### Genotyping for *Ss-unc-22* deletions

Primer sets used to amplify the regions around *Ss-unc-22* target sites, and regions 10-kb upstream and downstream of *Ss-unc-22* site #3, can be found in S13 Table. All deletion genotyping PCR reactions were performed with GoTaq G2 Flexi DNA Polymerase (Promega, Cat. # M7801) using the following thermocycler conditions: denature 95°C (2 min); PCR 95°C (30 s), 55°C (30 s), 72°C (1 min) x 35 cycles; final extension 72°C (5 min); 10°C (hold). All PCR products were resolved on ~1% agarose gels stained with GelRed (Biotium, Cat. # 41003) using a 1-kb marker (NEB, Cat. # N3232L). Quantification of PCR products shown in Fig 4B was performed with a ChemiDoc MP Imaging System using the Image Lab Version 5.1 Relative Quantity Tool. Individual control and *Ss-unc-22* site #3 bands from wild-type iL3s were randomly selected as reference bands. All other control and *Ss-unc-22* site #3 reactions were compared to the appropriate reference to determine relative quantity of PCR products. For all samples, 25 μL of PCR product were loaded on the gel and all samples were run on the same gel.

### Genotyping for *Ss-unc-22* and *Ss-tax-4* HDR

Primer sets used to test for HDR at *Ss-unc-22* and *Ss-tax-4* target sites can be found in S13 Table. PCR reactions for HDR of the repair template pEY09 at *Ss-unc-22* site #2 and HDR of the repair template pMLC39 at *Ss-tax-4* site #1 were performed with GoTaq G2 Flexi DNA Polymerase using the same thermocycler conditions outlined above, except for *Ss-tax-4* genotyping, where the extension time was 2 min. PCR products were resolved on ~1% agarose gels stained with GelRed with a 1-kb marker. 5’ and 3’ integration bands for *Ss-unc-22*, and 5’ integration bands for *Ss-tax-4*, were gel-extracted using the QIAquick Gel Extraction Kit (Qiagen, Cat. # 28704) and subcloned into pGEM-T Easy (Promega, Cat. # A1360) for sequencing. PCR reactions to test for ssODN incorporation at *Ss-unc-22* site #3 were performed with Platinum Taq DNA Polymerase (ThermoFisher Cat. # 10966018) using the same thermocycler conditions described for GoTaq. PCR products were resolved on ~1% agarose gels stained with GelRed using a 100-bp marker.

### Restriction enzyme digestion to test for ssODN incorporation

The primer set used to amplify the region around *Ss-unc-22* site #3 for ssODN EagI digest can be found in S13 Table. PCR cleanup on wild-type and *unc* iL3 pools was performed using the QIAquick PCR purification kit (Qiagen, Cat. # 28104). For each sample, ~1 μg of template DNA was digested with EagI-HF (NEB, Cat. # R3505S) at 37°C for 1 h. Digested products were resolved on ~1% agarose gels stained with GelRed using 1-kb and 100-bp markers.

### Illumina sequencing of *S*. *stercoralis*

Genomic DNA from populations of wild-type iL3s or *Ss-unc-22-*targeted F_1_ iL3s were collected as described above. 2x 150 paired-end Illumina libraries were prepared from 1 μg genomic DNA using the KAPA Library Preparation Kit with beads for size selection and sample cleanup (Kapa Biosystems, Cat. # KK8232). Libraries were sequenced on the Illumina HiSeq3000 platform using the HiSeq 3000/4000 PE Cluster Kit according to the manufacturer’s recommended protocol. Paired-end reads were mapped to the *S*. *stercoralis* reference genome using HISAT2 with the “—no-spliced-alignment” option [24,55]. To estimate read coverage, we performed a two-step approach. First, we computed per-site coverage using SAMtools for all sites that mapped to the reference. Second, to account for correlation between neighboring sites, we sampled 10,000 random sites to estimate coverage parameters under a negative-binomial distribution using custom Python and R scripts. The negative-binomial distribution is commonly used for modeling the random distribution of count data with an over-dispersion parameter and has been used in many bioinformatics pipelines to model read coverage [56]. We performed this second step to estimate genome-wide coverage parameters as well as parameters restricted to *Ss-unc-22* sites. To compute the probability of observing coverage depletion at a given *Ss-unc-22* target site by chance, we performed a one-tailed test under the *Ss-unc-22-*fitted negative binomial. To calculate indel frequency at *Ss-unc-22* site #3, we took the mapped reads generated above and ran CRISPRessoWGS and CRISPRessoCompare with default parameters [29].

### Fluorescence microscopy

*unc* F_1_ iL3s with a nicotine-twitching phenotype were transferred to a chemotaxis plate and screened for *mRFPmars* expression under a Leica M165 FC microscope. *unc* F_1_ iL3s expressing *mRFPmars* were mounted on a pad consisting of 5% Noble agar dissolved in ddH_2_O. Epifluorescence images were captured using a Zeiss AxioImager A2 microscope with an attached Zeiss Axiocam camera. Images were processed using Zeiss AxioVision software. After imaging, individual *unc* F_1_ iL3s were collected from agar pads and transferred to 5-6 μL of worm lysis buffer for HDR genotyping as described above.

### Germline transmission of *Ss-unc-22* mutations

A summary of *Ss-unc-22* germline transmission strategies can be found in S7 Table. To generate an ~50/50 mix of wild-type and *unc* F_1_ iL3s, free-living adult females were injected with RNP complex targeting *Ss-unc-22* site #3 with an ssODN. F_1_ iL3s were collected and a subset of them were screened in 1% nicotine assays to estimate the nicotine-twitching frequency; ~52% of iL3s contained putative *Ss-unc-22* mutations based solely on phenotypic observation in nicotine. The remaining F_1_ population was injected into gerbil hosts. To enrich for *unc* F_1_ iL3s, RNP injections targeting site #3 were carried out as described for the 50/50 mixed infection. F_1_ iL3s were collected and all nematodes were screened in 1% nicotine assays. Nicotine-twitching iL3s were selected, washed in ddH_2_O, and recovered from nicotine treatment overnight. Paralyzed iL3s were discarded. *unc* F_1_ iL3s recovered from nicotine were injected into gerbil hosts. In the control infection, wild-type iL3s were treated with nicotine to induce paralysis, washed in ddH_2_O, and recovered from nicotine treatment overnight. Recovered wild-type iL3s were injected into gerbil hosts. Feces from all of the host infection strategies were collected as described above. F_2_ and F_3_ nematodes were screened for *unc* phenotypes using the nicotine, swimming, and crawling assays described above.

### Statistical analysis

Statistical analysis was performed using standard statistical tests in GraphPad Prism Version 7.0. Deep-sequencing analysis was performed using custom Python and R scripts, and is described in detail above. The standard statistical tests used for all other experiments are described in the figure captions, and are also summarized below. For these experiments, the D’Agostino-Pearson omnibus normality test was first used to determine whether values came from a Gaussian distribution. If data were normally distributed, parametric tests were used; otherwise, non-parametric tests were used. A Mann-Whitney test or unpaired t-test with Welch’s correction was used to compare swimming and crawling behaviors in wild-type iL3s vs. *unc* iL3s (Figs 2, 6C and 6D). A chi-square test with Bonferroni correction or Fisher’s exact test was used to compare nicotine-induced twitching frequencies across genotypes or conditions (Figs 3 and 6B, S1, S2, S6 and S9 Figs). Depletion of *Ss-unc-22* site #3 in *unc* iL3s was quantified using a two-way ANOVA with Sidak’s post-test (Fig 4B).

## Supporting information

S1 Fig Nicotine induces twitching in *C*. *elegans unc-22* adults and dauers. Twitching frequency of *C*. *elegans* wild-type and *unc-22* adults and dauers. Twitching frequency differs for *C*. *elegans* wild-type and *unc-22* adults and dauers. *P<0.05, ***P<0.001, chi-square test with Bonferroni correction. n = 50–51 nematodes for each genotype and life stage.
(PDF)

S2 Fig Targeted mutagenesis in *S*. *ratti* using CRISPR-Cas9. (**A**) The *unc-22* gene of *S*. *ratti*. The *Sr-unc-22* gene structure depicted is based on the gene prediction from WormBase ParaSite [24,47]. The locations of the CRISPR target sites tested and predicted on-target activity scores are indicated [50]. Scale bar = 1 kb. (**B**) Twitching frequency of *S*. *ratti* wild-type iL3s and *Sr-unc-22-*targeted F_1_ iL3s following 1% nicotine exposure. For each condition, the *Sr-unc-22* target site and delivery method of CRISPR constructs are indicated. Twitching frequency of F_1_ iL3s for each target site differs from wild-type iL3s and from each other. *P<0.05, **P<0.01, ***P<0.001, chi-square test with Bonferroni correction. n = 267–544 iL3s for each condition.
(PDF)

S3 Fig Whole-genome sequencing reveals that *Ss-unc-22* sites #1 and #2 are not deleted when *Ss-unc-22* site #3 is targeted with CRISPR-Cas9. (**A-B**) Whole-genome sequencing coverage plots for *Ss-unc-22* site #1 (**A**) or site #2 (**B**) from populations of either *Ss-unc-22-*targeted F_1_ iL3s from P_0_ females injected with RNP complexes for site #3, or wild-type iL3s. A 4-kb window centered on the predicted cut sites is shown [24,47]. Black lines = average coverage depth by position (reads per base); red lines = average genome-wide coverage; blue lines = average coverage for the *Ss-unc-22* gene. Coverage around *Ss-unc-22* sites #1 and #2 is not depleted in *Ss-unc-22* libraries when *Ss-unc-22* site #3 is targeted (*P*>0.05; see Methods). Similarly, no coverage depletion is observed in the wild-type library (*P*>0.05; see Methods). For **B,** the gray shaded region represents significant depletion around *Ss-unc-22* site #3, which is only ~2.3 kb upstream of *Ss-unc-22* site #2. The arrow indicates that site #3 is upstream of the 4-kb window shown.
(PDF)

S4 Fig Whole-genome sequencing reveals that a distinct CRISPR target site is not deleted when *Ss-unc-22* site #3 is targeted. Whole-genome sequencing coverage plots for a selected control gene, *Ss-tax-4* (SSTP_0000981000) containing an unrelated predicted CRISPR target site. A 4-kb window centered on the predicted cut site is shown [24,47]. Black lines = average coverage depth by position (reads per base); red lines = average genome-wide coverage; blue lines = average coverage for the *Ss-tax-4* gene. Coverage around *Ss-tax-4* site #1 is not depleted in *Ss-unc-22* libraries when *Ss-unc-22* site #3 is targeted (*P*>0.05; see Methods). Similarly, no coverage depletion is observed in the wild-type library (*P*>0.05; see Methods).
(PDF)

S5 Fig CRISPR-Cas9-mediated deletions of *Ss-unc-22* site #3 do not disrupt nearby genomic loci. (**A**) The genomic region of *Ss-unc-22*. The gene structures of *Ss-unc-22*, and a downstream gene *Ss-rgr-1* (SSTP_0000032000), were based on the predictions from WormBase ParaSite [24,47]. Wild-type iL3s and *unc* F_1_ iL3s were genotyped for the *Ss-unc-22* site #3 target, 10 kb upstream of the target, and 10 kb downstream of the target using the primer sets indicated. Scale bar = 1 kb. (**B**) Representative gel of a wild-type iL3 and *unc* F_1_ iL3s from RNP injections at site #3. Genomic DNA from each iL3 was split into four reactions: ctrl. = control reaction amplifying 416 bp of the first exon of the *Ss-act-2* gene to confirm the presence of genomic DNA; u22 = reaction amplifying 660 bp around site #3; a = 10 kb upstream of site #3, b = 10 kb downstream of site #3. Genomic loci 10 kb upstream and downstream of site #3 are intact in *unc* F_1_ iL3s with putative homozygous deletions of *Ss-unc-22*. Size markers = 1 kb and 500 bp from top to bottom.
(PDF)

S6 Fig Addition of an HDR template improves *unc* F_1_ iL3 nicotine-twitching frequency. (**A**) The twitching frequency in *unc* F_1_ iL3s increases when a repair template containing *Ss-act-2*::*mRFPmars* is included in plasmid vector injections. ***P<0.001, Fisher’s exact test. n = 677–788 iL3s for each condition. (**B**) The twitching frequency in *unc* F_1_ iL3s increases when an ssODN is included in RNP injections. ***P<0.001, Fisher’s exact test. n = 619–830 iL3s for each condition.
(PDF)

S7 Fig Sequencing for HDR with the *Ss-act-2*::*mRFPmars* repair template at *Ss-unc-22* site #2. (**A-B**) Sequencing results showing insertion of the repair template spanning the 5’ border of the integrated cassette (**A**) or the 3’ border of the integrated cassette (**B**). The relevant regions of *Ss-unc-22*, the repair template, and the primer binding sites are highlighted and color-coded to match the schematic shown in Fig 5A.
(PDF)

S8 Fig ssODNs are not templates for HDR at *Ss-unc-22*. (**A**) Strategy for ssODN-mediated HDR of CRISPR mutations at *Ss-unc-22* site #3. RNP complexes targeting site #3 mixed with ssODN were injected into free-living adult females. *unc* F_1_ iL3s that displayed the twitching phenotype were selected as candidates for HDR and were genotyped for ssODN incorporation using the primer sets indicated. HA = homology arm. (**B**) The ssODN sequence for *Ss-unc-22* site #3. The ssODN contains stop codons in all reading frames, an EagI restriction site, and the sequence for the T7 primer flanked on either end by 5’ and 3’ homology arms that match the genomic DNA upstream and downstream of site #3. (**C**) The ssODN failed to incorporate at site #3 by PCR. Top gel: lane 1 = control to confirm primer R4 can amplify from *S*. *stercoralis* genomic DNA and is present in the reaction, lane 2 = control to confirm primer T7 can amplify from a plasmid vector and is present in the reaction, lanes 3–6 = reactions with primers T7 x R4 show no evidence for ssODN incorporation from pools of 10–15 wild-type iL3s or *unc* F_1_ iL3s. Bottom gel: lanes 2–10 = reactions with primers T7 x R4 show no evidence for ssODN incorporation from an individual wild-type iL3 or individual *unc* F_1_ iL3s. (**D**) The ssODN failed to incorporate at site #3 by EagI digest. Lanes 1–2 = EagI digest controls with plasmid vector, lanes 3–4 = EagI digest from >5,000 wild-type iL3s (population) or 10–15 wild-type iL3s (pool), lanes 5–8 = EagI digest from a mixed population of >5,000 twitching *unc* F_1_ iL3s and not twitching wild-type iL3s (population), or 10–15 twitching *unc* F_1_ iL3s (pools). Successful ssODN incorporation at *Ss-unc-22* site #3 would be expected to produce ~300 bp EagI digestion products. No digestion products were observed. Size markers = 100-bp ladder for **C**, or 100-bp and 1-kb ladder for **D.**
(PDF)

S9 Fig Removing Cas9 from injections abolishes *Ss-unc-22* targeted mutagenesis. (**A**) The nicotine-twitching phenotype was not observed in F_1_ iL3s when the plasmid vector for the expression of Cas9 was excluded from the injection mix. ***P<0.001, Fisher’s exact test. n = 346–788 iL3s for each condition. (**B**) The nicotine-twitching phenotype was not observed in F_1_ iL3s when Cas9 protein was excluded from RNP complex assembly. ***P<0.001, Fisher’s exact test. n = 353–1,284 iL3s for each condition.
(PDF)

S10 Fig CRISPR-mediated homology-directed repair of *Ss-tax-4*. (**A**) The *tax-4* genes of *C*. *elegans* and *S*. *stercoralis*. The *Ss-tax-4* gene structure is based on the gene prediction from WormBase Parasite [24,47]. The CRISPR target site tested and the on-target activity score are indicated [50]. Scale bars = 1 kb. (**B**) Strategy for HDR at *Ss-tax-4* target site #1. F_1_ iL3s that displayed red fluorescence were selected as candidates for HDR and were genotyped using the primer sets indicated. The 5’ integration primers only amplify following successful integration of *Ss-act-2*::*mRFPmars* into site #1. HA = homology arm. (**C**) Representative genotypes of F_1_ iL3s expressing *mRFPmars* collected from *Ss-tax-*4-CRISPR microinjected females. Genomic DNA from individual iL3s was split into two reactions: wt = reaction for the wild-type locus of site #1; 5’ = reaction for insertion of the 5’ border of the integrated cassette. For genotypes: array = red iL3s that showed no evidence of integration; int. = red *Ss-tax-4* iL3s with successful HDR. Asterisks indicate iL3s that were sequenced for 5’ integration at the *Ss-tax-4* locus. Size markers = 1-kb ladder. The gel was cropped for conciseness of presentation. (**D**) Sequencing results showing insertion of the repair template; the sequence spans the 5’ border of the integrated cassette. The relevant regions of the *Ss-tax-4* repair template are highlighted and color-coded to match the schematic shown in **B**.
(PDF)

S1 Table Summary of CRISPR-Cas9 targeting efficiency for *Ss-unc-22*. Results for combined nicotine assay data presented in Fig 3B. The estimated number of F_1_ iL3s collected from each injection experiment was based on the average number of iL3s per injected adult calculated in S14 Table.
(PDF)

S2 Table Summary of CRISPR-Cas9 targeting efficiency for *Sr-unc-22*. Results for combined nicotine assay data presented in S2 Fig. The estimated number of F_1_ iL3s collected from each injection experiment was based on the average number of iL3s per injected adult calculated in S14 Table.
(PDF)

S3 Table Summary of *Ss-unc-22* deletions. Individual wild-type iL3s, and *unc* F_1_ iL3s that displayed a nicotine-twitching phenotype, were collected and genomic DNA was prepared. For each iL3, the region around the *Ss-unc-22* target was PCR-amplified along with a control reaction from a different contig than *Ss-unc-22*, as shown in Fig 4A. Instances where the *Ss-unc-22* target failed to amplify but the control reaction was present were considered putative homozygous deletions of *Ss-unc-22*.
(PDF)

S4 Table Summary of sample preparation for *Ss-unc-22* whole-genome sequencing. *S*. *stercoralis* free-living adults were injected with RNP complexes targeting *Ss-unc-22* site #3, including an ssODN, and F_1_ iL3s were collected. A subset of F_1_ iL3s was screened for nicotine-twitching frequency to estimate the *Ss-unc-22* mutation rate and the remaining population was split in two for iL3 lysis, genomic DNA extraction, and Illumina library preparation. Wild-type iL3s were also screened for nicotine-twitching frequency and an Illumina library was prepared from a wild-type population in parallel with the *Ss-unc-22* libraries.
(PDF)

S5 Table Summary of CRISPR-Cas9 targeting efficiency for *Ss-unc-22* with HDR constructs. Results for combined nicotine assay data presented in S6 Fig. The estimated number of F_1_ iL3s collected from each injection experiment was based on the average number of iL3s per injected adult calculated in S14 Table. n.a. = not available; the number of free-living adults injected was not recorded for this experiment.
(PDF)

S6 Table Summary of HDR in *S*. *stercoralis*. Free-living adult females were injected with CRISPR-Cas9 plasmid vectors and repair template targeting either *Ss-unc-22* site #2 or *Ss-tax-4* site #1. Individual F_1_ iL3s expressing *mRFPmars* were genotyped for repair template integration as shown in Fig 5 and S10 Fig.
(PDF)

S7 Table Host infection and germline transmission of *Ss-unc-22* mutations. Summary of host passage strategies for wild-type control, 50/50 mixed *unc* and wild-type, and *unc*-enriched infections. Results for total recovery of F_2_ and F_3_ progeny for each infection strategy, and the combined nicotine-assay data presented in Fig 6B, are provided. n.a. = not available; the number of iL3s recovered was not recorded for these experiments.
(PDF)

S8 Table Plasmid vectors for *Strongyloides* CRISPR-Cas9. pPV540, provided by Dr. James Lok, was modified from pPV402, which is described in Shao *et al*. 2012 [45]. pEY09 and pMLC39 were modified from pAJ50, which is described in Junio *et al*. 2008 [31].
(PDF)

S9 Table *Strongyloides* CRISPR target sequences. The PAM for each target sequence is underlined. Note that each target sequence contains guanine residues in the 1^st^, 19^th^, and 20^th^ positions (GN(17)GG), as recommended in Farboud *et al*. 2015 [49].
(PDF)

S10 Table Plasmid vector injection mixes for *Strongyloides* CRISPR-Cas9. Injection mixes introduced into *Strongyloides* free-living adult females were limited to a maximum final DNA concentration of 100 ng/μL, as previously described in Junio *et al*. 2008 [31].
(PDF)

S11 Table *Ss-unc-22* crRNA sequences. The first 20 nucleotides of each crRNA match the genomic DNA of the CRISPR target site indicated. The remaining nucleotides are the *Streptococcus pyogenes* repeat sequence and are identical for all three crRNAs shown.
(PDF)

S12 Table RNP injection mixes for *Ss-unc-22* CRISPR-Cas9. RNP injection mixes were based on the injection mixes described in Paix *et al*. 2015 [20].
(PDF)

S13 Table Primer sets used in this study. (PDF)

S14 Table The average number of F_1_ iL3s collected per microinjected free-living adult female for *S*. *stercoralis* and *S*. *ratti*. Injected free-living adult females were reared on host feces based on the observation that it results in higher reproductive output relative to other standard culturing methods [53]. iL3s were collected using a Baermann apparatus and all F_1_ progeny were counted to calculate the average number of iL3s per injected adult.
(PDF)

S1 Video An *S*. *stercoralis* wild-type iL3 swimming in dH_2_O. Scale bar = 200 μm.
(MOV)

S2 Video An *S*. *stercoralis unc* F_1_ iL3 swimming in dH_2_O. Scale bar = 200 μm.
(MOV)

S3 Video An *S*. *stercoralis* wild-type iL3 crawling on agar. Scale bar = 1 mm.
(MOV)

S4 Video An *S*. *stercoralis unc* F_1_ iL3 crawling on agar. Scale bar = 1 mm.
(MOV)

S5 Video An *S*. *stercoralis* wild-type iL3 paralyzed after 8 min in 1% nicotine. Scale bar = 100 μm.
(MOV)

S6 Video An *S*. *stercoralis unc* F_1_ iL3 twitching after 8 min in 1% nicotine. Scale bar = 100 μm.
(MOV)

S7 Video An *S*. *stercoralis unc* F_2_ or F_3_ iL3 twitching after 8 min in 1% nicotine. Scale bar = 100 μm.
(MOV)

S8 Video An *S*. *stercoralis* wild-type free-living adult female paralyzed after 8 min in 1% nicotine. Scale bar = 100 μm.
(MOV)

S9 Video An *S*. *stercoralis unc* F_2_ free-living adult female twitching after 8 min in 1% nicotine. Scale bar = 100 μm.
(MOV)
