# Supplementary figures and images for: Targeted mutagenesis in a human-parasitic nematode
Source: PLoS Pathog. 2017 Oct 10;13(10):e1006675. doi: 10.1371/journal.ppat.1006675 (PMC5650185; doi:10.1371/journal.ppat.1006675)

S1 Fig.

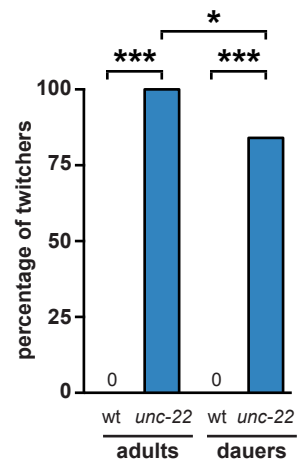

Supplement: S1 Fig — Twitching frequency of C. elegans wild-type and unc-22 adults and dauers. Twitching frequency differs for C. elegans wild-type and unc-22 adults and dauers. *P<0.05, ***P<0.001, chi-square test with Bonferroni correction. n = 50–51 nematodes for each genotype and life stage. (PDF) [file ppat.1006675.s001.pdf]

A

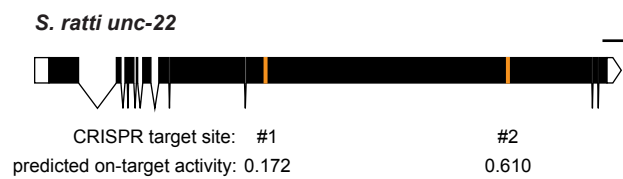

B

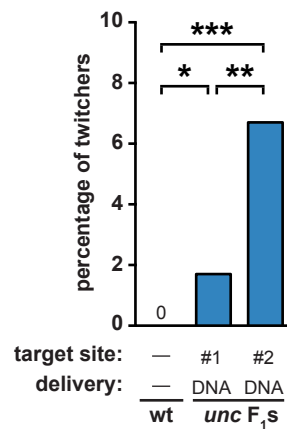

Supplement: S2 Fig — (A) The unc-22 gene of S. ratti. The Sr-unc-22 gene structure depicted is based on the gene prediction from WormBase ParaSite [24,47]. The locations of the CRISPR target sites tested and predicted on-target activity scores are indicated [50]. Scale bar = 1 kb. (B) Twitching frequency of S. ratti wild-type iL3s and Sr-unc-22-targeted F1 iL3s following 1% nicotine exposure. For each condition, the Sr-unc-22 target site and delivery method of CRISPR constructs are indicated. Twitching frequency of F1 iL3s for each target site differs from wild-type iL3s and from each other. *P<0.05, **P<0.01, ***P<0.001, chi-square test with Bonferroni correction. n = 267–544 iL3s for each condition. (PDF) [file ppat.1006675.s002.pdf]

A

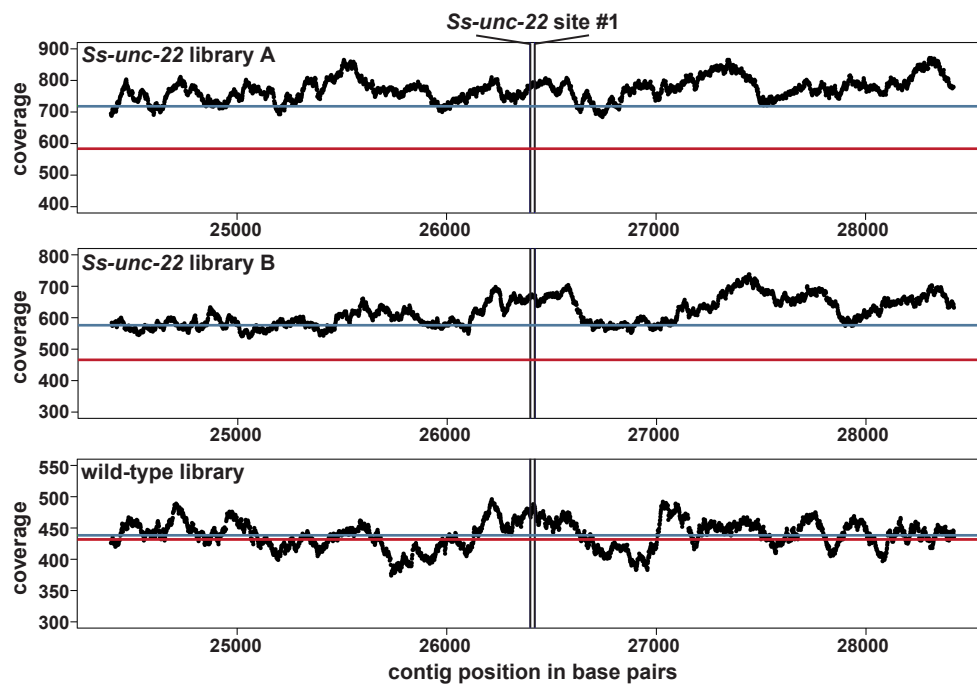

B

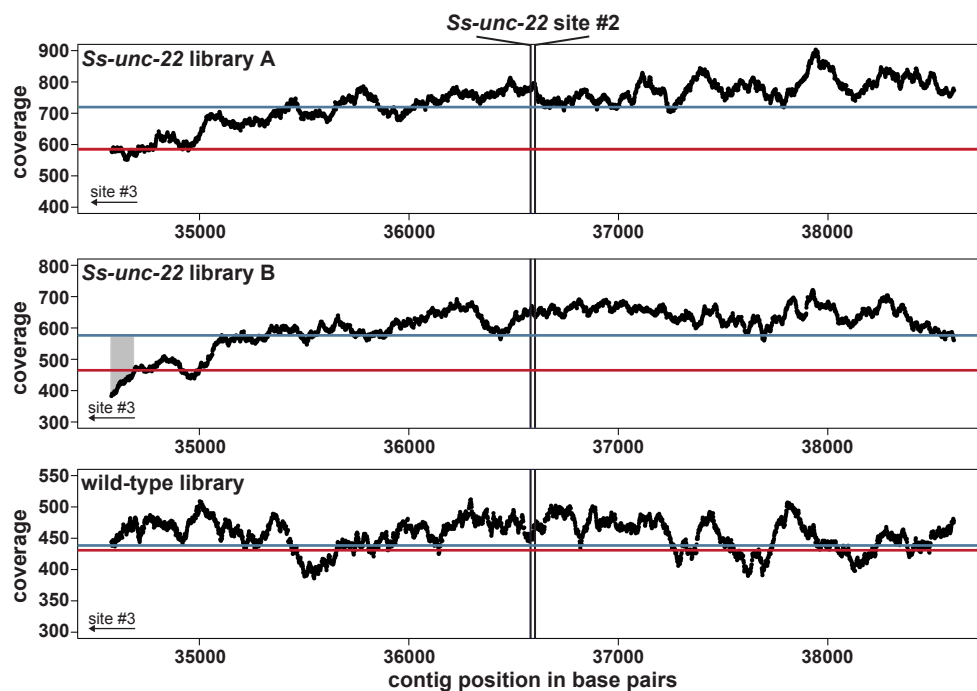

Supplement: S3 Fig — (A-B) Whole-genome sequencing coverage plots for Ss-unc-22 site #1 (A) or site #2 (B) from populations of either Ss-unc-22-targeted F1 iL3s from P0 females injected with RNP complexes for site #3, or wild-type iL3s. A 4-kb window centered on the predicted cut sites is shown [24,47]. Black lines = average coverage depth by position (reads per base); red lines = average genome-wide coverage; blue lines = average coverage for the Ss-unc-22 gene. Coverage around Ss-unc-22 sites #1 and #2 is not depleted in Ss-unc-22 libraries when Ss-unc-22 site #3 is targeted (P>0.05; see Methods). Similarly, no coverage depletion is observed in the wild-type library (P>0.05; see Methods). For B, the gray shaded region represents significant depletion around Ss-unc-22 site #3, which is only ~2.3 kb upstream of Ss-unc-22 site #2. The arrow indicates that site #3 is upstream of the 4-kb window shown. (PDF) [file ppat.1006675.s003.pdf]

S4 Fig.

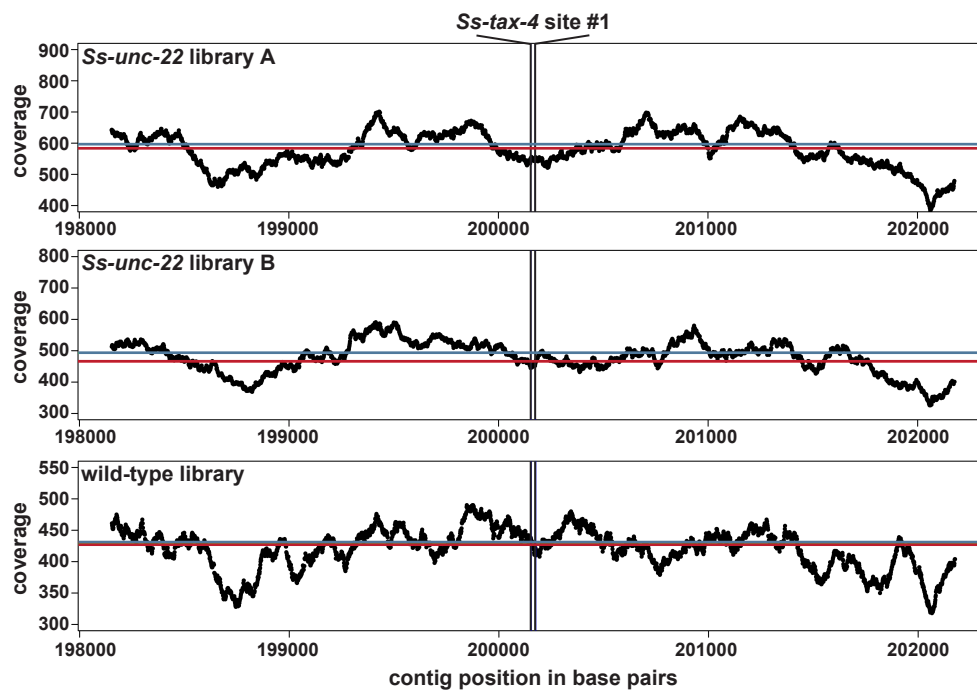

Supplement: S4 Fig — Whole-genome sequencing coverage plots for a selected control gene, Ss-tax-4 (SSTP_0000981000) containing an unrelated predicted CRISPR target site. A 4-kb window centered on the predicted cut site is shown [24,47]. Black lines = average coverage depth by position (reads per base); red lines = average genome-wide coverage; blue lines = average coverage for the Ss-tax-4 gene. Coverage around Ss-tax-4 site #1 is not depleted in Ss-unc-22 libraries when Ss-unc-22 site #3 is targeted (P>0.05; see Methods). Similarly, no coverage depletion is observed in the wild-type library (P>0.05; see Methods). (PDF) [file ppat.1006675.s004.pdf]

A

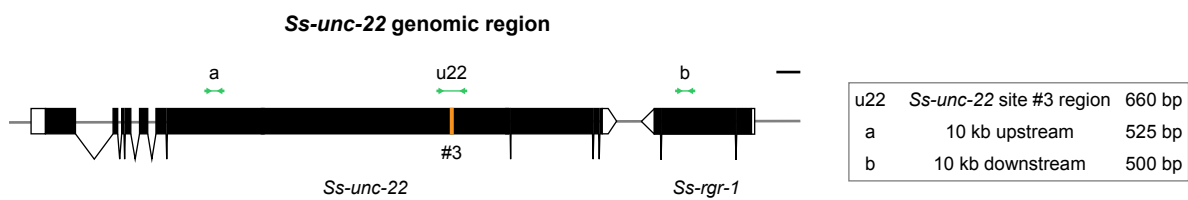

B

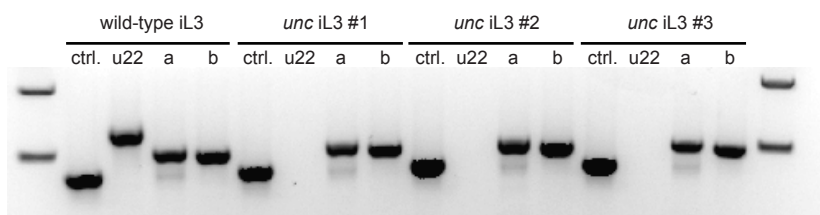

Supplement: S5 Fig — (A) The genomic region of Ss-unc-22. The gene structures of Ss-unc-22, and a downstream gene Ss-rgr-1 (SSTP_0000032000), were based on the predictions from WormBase ParaSite [24,47]. Wild-type iL3s and unc F1 iL3s were genotyped for the Ss-unc-22 site #3 target, 10 kb upstream of the target, and 10 kb downstream of the target using the primer sets indicated. Scale bar = 1 kb. (B) Representative gel of a wild-type iL3 and unc F1 iL3s from RNP injections at site #3. Genomic DNA from each iL3 was split into four reactions: ctrl. = control reaction amplifying 416 bp of the first exon of the Ss-act-2 gene to confirm the presence of genomic DNA; u22 = reaction amplifying 660 bp around site #3; a = 10 kb upstream of site #3, b = 10 kb downstream of site #3. Genomic loci 10 kb upstream and downstream of site #3 are intact in unc F1 iL3s with putative homozygous deletions of Ss-unc-22. Size markers = 1 kb and 500 bp from top to bottom. (PDF) [file ppat.1006675.s005.pdf]

**A**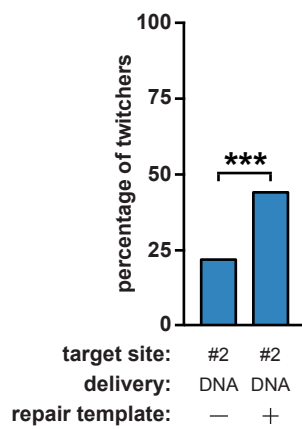**B**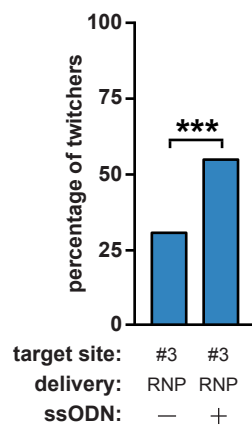

Supplement: S6 Fig — (A) The twitching frequency in unc F1 iL3s increases when a repair template containing Ss-act-2::mRFPmars is included in plasmid vector injections. ***P<0.001, Fisher’s exact test. n = 677–788 iL3s for each condition. (B) The twitching frequency in unc F1 iL3s increases when an ssODN is included in RNP injections. ***P<0.001, Fisher’s exact test. n = 619–830 iL3s for each condition. (PDF) [file ppat.1006675.s006.pdf]

A

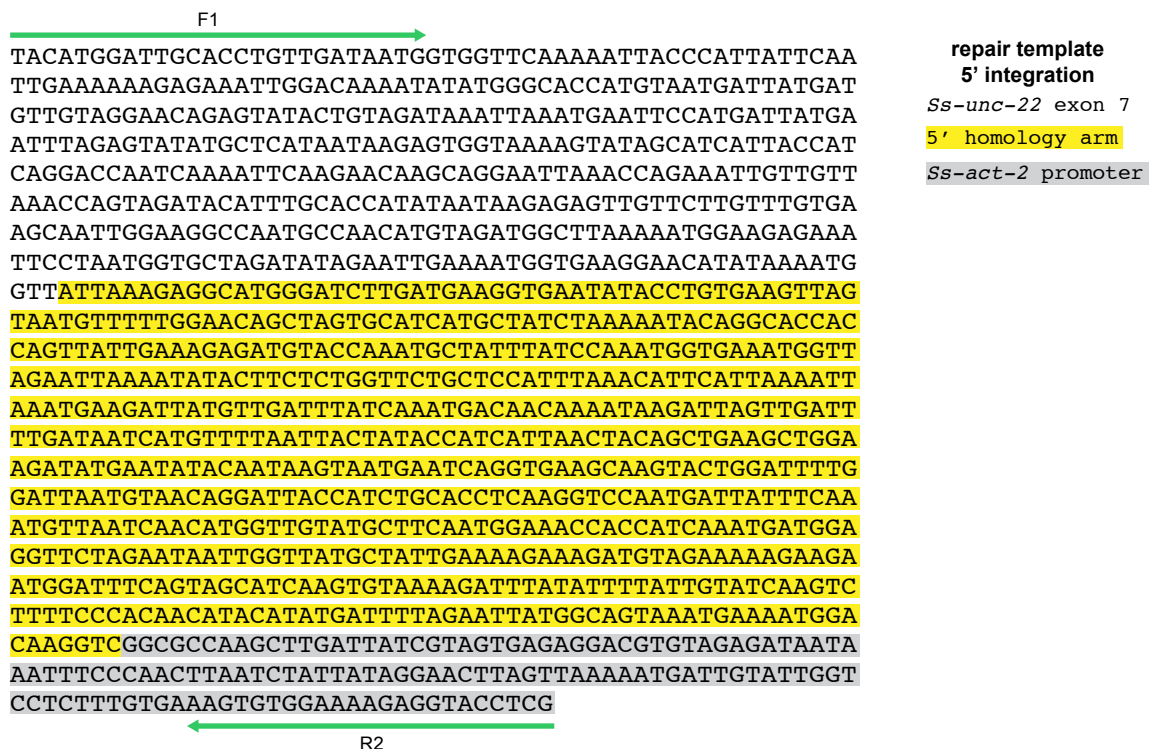

B

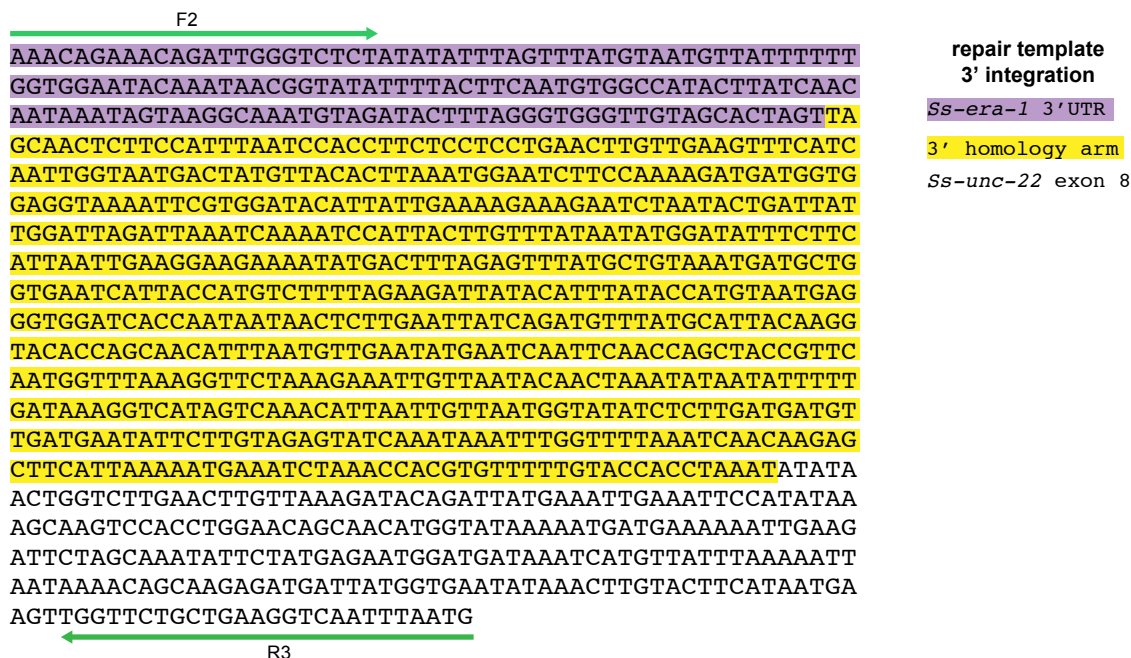

Supplement: S7 Fig — (A-B) Sequencing results showing insertion of the repair template spanning the 5’ border of the integrated cassette (A) or the 3’ border of the integrated cassette (B). The relevant regions of Ss-unc-22, the repair template, and the primer binding sites are highlighted and color-coded to match the schematic shown in Fig 5A. (PDF) [file ppat.1006675.s007.pdf]

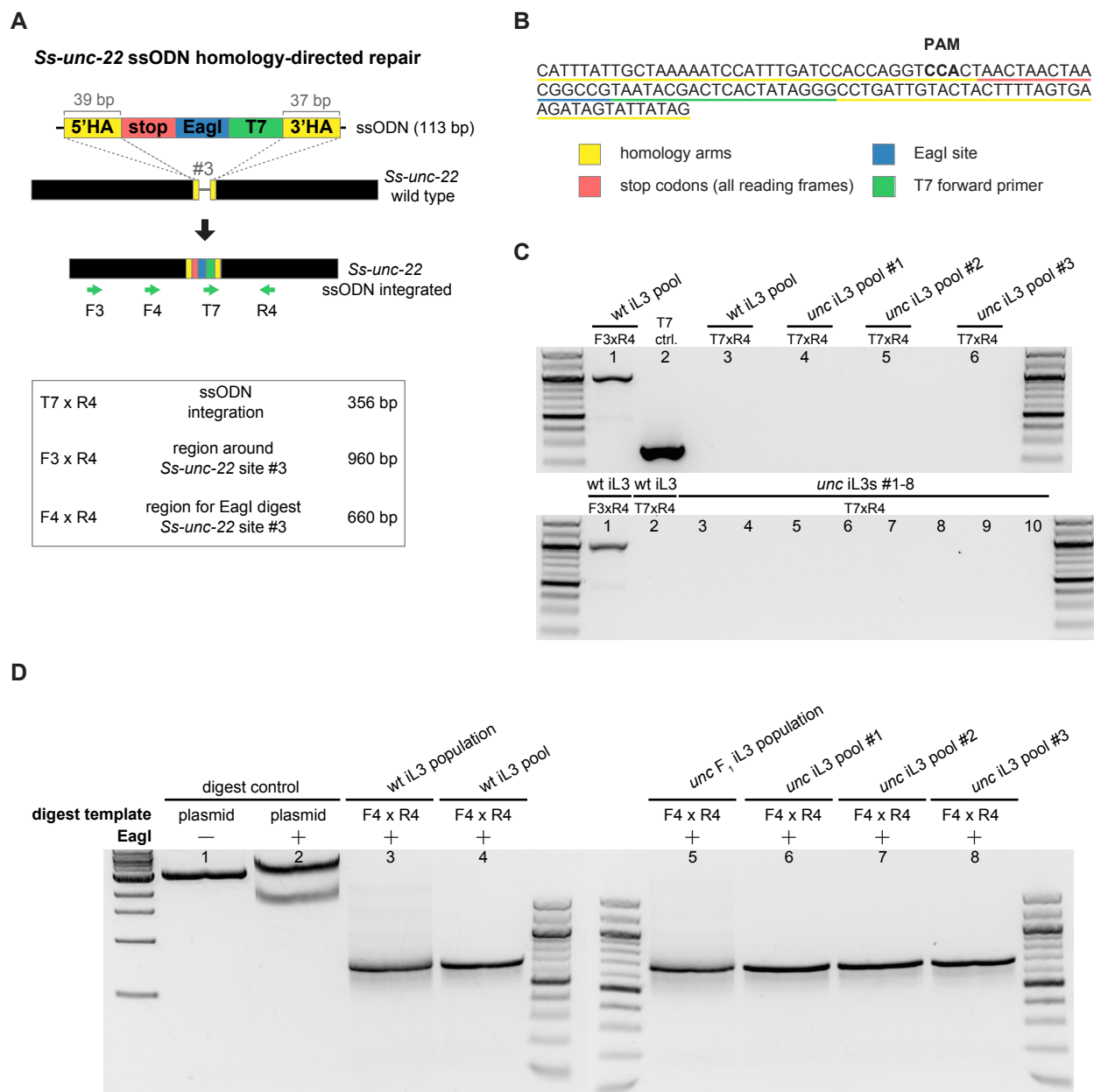

Supplement: S8 Fig — (A) Strategy for ssODN-mediated HDR of CRISPR mutations at Ss-unc-22 site #3. RNP complexes targeting site #3 mixed with ssODN were injected into free-living adult females. unc F1 iL3s that displayed the twitching phenotype were selected as candidates for HDR and were genotyped for ssODN incorporation using the primer sets indicated. HA = homology arm. (B) The ssODN sequence for Ss-unc-22 site #3. The ssODN contains stop codons in all reading frames, an EagI restriction site, and the sequence for the T7 primer flanked on either end by 5’ and 3’ homology arms that match the genomic DNA upstream and downstream of site #3. (C) The ssODN failed to incorporate at site #3 by PCR. Top gel: lane 1 = control to confirm primer R4 can amplify from S. stercoralis genomic DNA and is present in the reaction, lane 2 = control to confirm primer T7 can amplify from a plasmid vector and is present in the reaction, lanes 3–6 = reactions with primers T7 x R4 show no evidence for ssODN incorporation from pools of 10–15 wild-type iL3s or unc F1 iL3s. Bottom gel: lanes 2–10 = reactions with primers T7 x R4 show no evidence for ssODN incorporation from an individual wild-type iL3 or individual unc F1 iL3s. (D) The ssODN failed to incorporate at site #3 by EagI digest. Lanes 1–2 = EagI digest controls with plasmid vector, lanes 3–4 = EagI digest from >5,000 wild-type iL3s (population) or 10–15 wild-type iL3s (pool), lanes 5–8 = EagI digest from a mixed population of >5,000 twitching unc F1 iL3s and not twitching wild-type iL3s (population), or 10–15 twitching unc F1 iL3s (pools). Successful ssODN incorporation at Ss-unc-22 site #3 would be expected to produce ~300 bp EagI digestion products. No digestion products were observed. Size markers = 100-bp ladder for C, or 100-bp and 1-kb ladder for D. (PDF) [file ppat.1006675.s008.pdf]

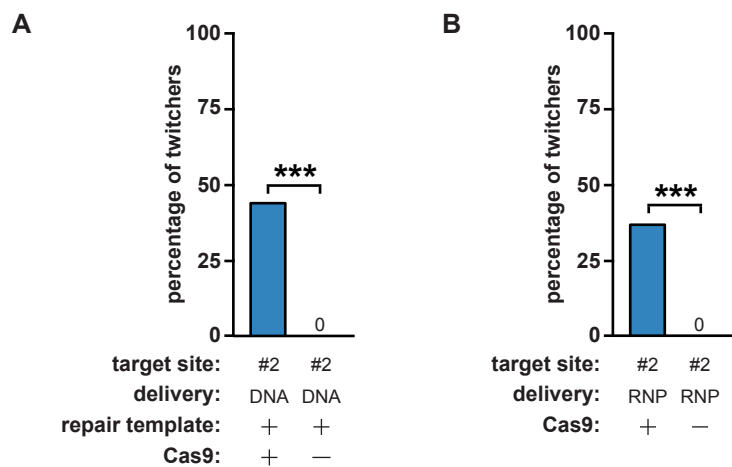

Supplement: S9 Fig — (A) The nicotine-twitching phenotype was not observed in F1 iL3s when the plasmid vector for the expression of Cas9 was excluded from the injection mix. ***P<0.001, Fisher’s exact test. n = 346–788 iL3s for each condition. (B) The nicotine-twitching phenotype was not observed in F1 iL3s when Cas9 protein was excluded from RNP complex assembly. ***P<0.001, Fisher’s exact test. n = 353–1,284 iL3s for each condition. (PDF) [file ppat.1006675.s009.pdf]
