# Supplementary material for: Targeted mutagenesis in a human-parasitic nematode
Source: PLoS Pathog. 2017 Oct 10;13(10):e1006675. doi: 10.1371/journal.ppat.1006675 (PMC5650185; doi:10.1371/journal.ppat.1006675)
Supplement: S10 Fig — (A) The tax-4 genes of C. elegans and S. stercoralis. The Ss-tax-4 gene structure is based on the gene prediction from WormBase Parasite [24,47]. The CRISPR target site tested and the on-target activity score are indicated [50]. Scale bars = 1 kb. (B) Strategy for HDR at Ss-tax-4 target site #1. F1 iL3s that displayed red fluorescence were selected as candidates for HDR and were genotyped using the primer sets indicated. The 5’ integration primers only amplify following successful integration of Ss-act-2::mRFPmars into site #1. HA = homology arm. (C) Representative genotypes of F1 iL3s expressing mRFPmars collected from Ss-tax-4-CRISPR microinjected females. Genomic DNA from individual iL3s was split into two reactions: wt = reaction for the wild-type locus of site #1; 5’ = reaction for insertion of the 5’ border of the integrated cassette. For genotypes: array = red iL3s that showed no evidence of integration; int. = red Ss-tax-4 iL3s with successful HDR. Asterisks indicate iL3s that were sequenced for 5’ integration at the Ss-tax-4 locus. Size markers = 1-kb ladder. The gel was cropped for conciseness of presentation. (D) Sequencing results showing insertion of the repair template; the sequence spans the 5’ border of the integrated cassette. The relevant regions of the Ss-tax-4 repair template are highlighted and color-coded to match the schematic shown in B. (PDF) [file ppat.1006675.s010.pdf]

A

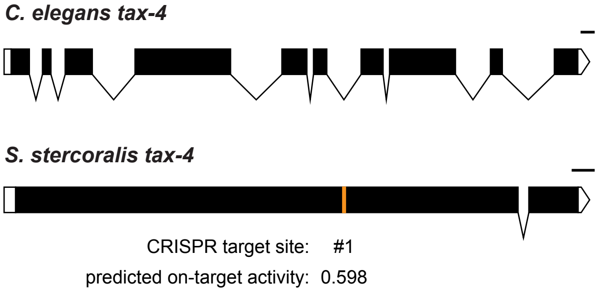

B

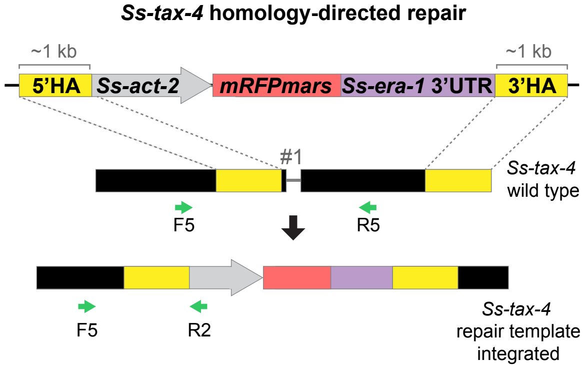

F5 x R5	wild type	1,822 bp
F5 x R2	5' integration	1,350 bp

C

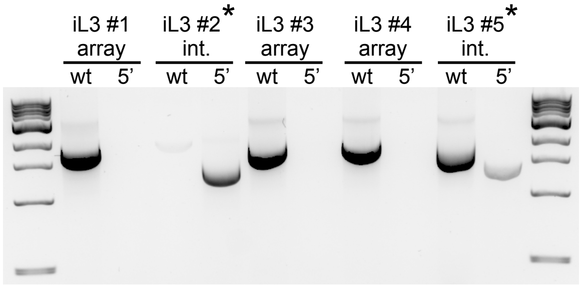

D

repair template 5' integration

Ss-tax-4 exon 1

5' homology arm

```
TATTAAAGTCACAAGTGTCAATTGAACCAATAACTAATAA
TCTTTCTAATAATAATTCTATGTCTGGTAATAGGCCAATG
ACTATGGATACCAAGAATAAAGAAAATGGTAATAAGATGA
ATGAGGGTCAAAAAGTTCCTCCAATAGTTAAAATATCTGT
TCCAGGGACTGTTAATGATCACGACTCTGAGTTGACTGAT
TTAGATCATGATAATGGAATTGATGTTGATGATACTATTG
TTATACAAAAAAATGACTCACCACCTTTAAGGGATAATTC
TAATTCTTCAAAAATGCCAAAGTCCCCAAAACAAAATGAG
GAAGTTAAAAA......
```
