# Supplementary material for: Targeted mutagenesis in a human-parasitic nematode
Source: PLoS Pathog. 2017 Oct 10;13(10):e1006675. doi: 10.1371/journal.ppat.1006675 (PMC5650185; doi:10.1371/journal.ppat.1006675)
Supplement: S1 Table — Results for combined nicotine assay data presented in Fig 3B. The estimated number of F1 iL3s collected from each injection experiment was based on the average number of iL3s per injected adult calculated in S14 Table. (PDF) [file ppat.1006675.s011.pdf]

S1 Table. Summary of CRISPR-Cas9 targeting efficiency for *Ss-unc-22*. Results for combined nicotine assay data presented in Fig 3B. The estimated number of F_1 iL3s collected from each injection experiment was based on the average number of iL3s per injected adult calculated in S14 Table.

target	delivery	injection experiment	# free-living adults injected (P_0)	estimated # F_1 iL3s collected	# iL3s screened	# twitching (%)
wild type	—	—	—	—	**1,314**	**2 (0.15%)**
1	DNA	a	29	~957	254	59 (23%)
		b	31	~1,023	192	19 (10%)
			**60**	**~1,980**	**446**	**78 (17%)**
1	RNP	a	27	~891	473	63 (13%)
		b	22	~726	435	82 (19%)
			**49**	**~1,617**	**908**	**145 (16%)**
2	DNA	a	31	~1,023	222	56 (25%)
		b	30	~990	123	20 (16%)
		c	20	~660	332	70 (21%)
			**81**	**~2,673**	**677**	**146 (22%)**
2	RNP	a	27	~891	668	201 (30%)
		b	22	~726	362	153 (42%)
		c	25	~825	254	116 (46%)
			**74**	**~2,442**	**1,284**	**470 (37%)**
3	DNA	a	24	~792	368	88 (24%)
		b	20	~660	235	80 (34%)
			**44**	**~1,452**	**603**	**168 (28%)**
3	RNP	a	33	~1,089	191	70 (37%)
		b	28	~924	428	118 (28%)
			**61**	**~2,013**	**619**	**188 (30%)**
