# Supplementary material for: Targeted mutagenesis in a human-parasitic nematode
Source: PLoS Pathog. 2017 Oct 10;13(10):e1006675. doi: 10.1371/journal.ppat.1006675 (PMC5650185; doi:10.1371/journal.ppat.1006675)
Supplement: S2 Table — Results for combined nicotine assay data presented in S2 Fig. The estimated number of F1 iL3s collected from each injection experiment was based on the average number of iL3s per injected adult calculated in S14 Table. (PDF) [file ppat.1006675.s012.pdf]

S2 Table. Summary of CRISPR-Cas9 targeting efficiency for *Sr-unc-22*. Results for combined nicotine assay data presented in S2 Fig. The estimated number of F_1 iL3s collected from each injection experiment was based on the average number of iL3s per injected adult calculated in S14 Table.

**target**	**delivery**	**injection experiment**	**# free-living adults injected (P_0)**	**estimated # F_1 iL3s collected**	**# iL3s screened**	**# twitching (%)**
wild type	—	—	—	—	**544**	**0 (0%)**
1	DNA	a	32	~800	196	2 (1%)
		b	30	~750	101	3 (3%)
			**62**	**~1,550**	**297**	**5 (2%)**
2	DNA	a	30	~750	110	9 (8%)
		b	28	~700	157	9 (6%)
			**58**	**~1,450**	**267**	**18 (7%)**
