# Supplementary material for: Targeted mutagenesis in a human-parasitic nematode
Source: PLoS Pathog. 2017 Oct 10;13(10):e1006675. doi: 10.1371/journal.ppat.1006675 (PMC5650185; doi:10.1371/journal.ppat.1006675)
Supplement: S3 Table — Individual wild-type iL3s, and unc F1 iL3s that displayed a nicotine-twitching phenotype, were collected and genomic DNA was prepared. For each iL3, the region around the Ss-unc-22 target was PCR-amplified along with a control reaction from a different contig than Ss-unc-22, as shown in Fig 4A. Instances where the Ss-unc-22 target failed to amplify but the control reaction was present were considered putative homozygous deletions of Ss-unc-22. (PDF) [file ppat.1006675.s013.pdf]

S3 Table. Summary of *Ss-unc-22* deletions. Individual wild-type iL3s, and *unc* F_1 iL3s that displayed a nicotine-twitching phenotype, were collected and genomic DNA was prepared. For each iL3, the region around the *Ss-unc-22* target was PCR-amplified along with a control reaction from a different contig than *Ss-unc-22*, as shown in Fig 4A. Instances where the *Ss-unc-22* target failed to amplify but the control reaction was present were considered putative homozygous deletions of *Ss-unc-22*.

target	delivery	repair	# iL3s genotyped	# putative *Ss-unc-22* -/- deletions (%)
1 (wild type)	—	—	38	**0 (0%)**
1	RNP	—	36	**0 (0%)**
2 (wild type)	—	—	76	**0 (0%)**
2	RNP	—	54	**1 (1.9%)**
3 (wild type)	—	—	46	**0 (0%)**
3	RNP	—	18	**1 (5.6%)**
3	RNP	ssODN	119	**6 (5%)**
