# Supplementary material for: Targeted mutagenesis in a human-parasitic nematode
Source: PLoS Pathog. 2017 Oct 10;13(10):e1006675. doi: 10.1371/journal.ppat.1006675 (PMC5650185; doi:10.1371/journal.ppat.1006675)
Supplement: S4 Table — S. stercoralis free-living adults were injected with RNP complexes targeting Ss-unc-22 site #3, including an ssODN, and F1 iL3s were collected. A subset of F1 iL3s was screened for nicotine-twitching frequency to estimate the Ss-unc-22 mutation rate and the remaining population was split in two for iL3 lysis, genomic DNA extraction, and Illumina library preparation. Wild-type iL3s were also screened for nicotine-twitching frequency and an Illumina library was prepared from a wild-type population in parallel with the Ss-unc-22 libraries. (PDF) [file ppat.1006675.s014.pdf]

S4 Table. Summary of sample preparation for *Ss-unc-22* whole-genome sequencing. *S. stercoralis* free-living adults were injected with RNP complexes targeting *Ss-unc-22* site #3, including an ssODN, and F_1 iL3s were collected. A subset of F_1 iL3s was screened for nicotine-twitching frequency to estimate the *Ss-unc-22* mutation rate and the remaining population was split in two for iL3 lysis, genomic DNA extraction, and Illumina library preparation. Wild-type iL3s were also screened for nicotine-twitching frequency and an Illumina library was prepared from a wild-type population in parallel with the *Ss-unc-22* libraries.

target	delivery	repair	# free-living adults injected (P_0)	estimated # iL3s collected	# iL3s screened in 1% nicotine	% iL3s twitching in 1% nicotine	# WGS libraries prepared
3	RNP	ssODN	280	~9,240	182	40.7%	2 (*Ss-unc-22* A + B)
—	—	—	—	~5,000	154	0%	1 (wild type)
