# Supplementary material for: Targeted mutagenesis in a human-parasitic nematode
Source: PLoS Pathog. 2017 Oct 10;13(10):e1006675. doi: 10.1371/journal.ppat.1006675 (PMC5650185; doi:10.1371/journal.ppat.1006675)
Supplement: S5 Table — Results for combined nicotine assay data presented in S6 Fig. The estimated number of F1 iL3s collected from each injection experiment was based on the average number of iL3s per injected adult calculated in S14 Table. n.a. = not available; the number of free-living adults injected was not recorded for this experiment. (PDF) [file ppat.1006675.s015.pdf]

S5 Table. Summary of CRISPR-Cas9 targeting efficiency for *Ss-unc-22* with HDR constructs. Results for combined nicotine assay data presented in S6 Fig. The estimated number of F_1 iL3s collected from each injection experiment was based on the average number of iL3s per injected adult calculated in S14 Table. n.a. = not available; the number of free-living adults injected was not recorded for this experiment.

target	delivery	repair	injection experiment	# free-living adults injected (P_0)	estimated # F_1 iL3s collected	# F_1 iL3s screened	# twitching (%)
2	DNA	pEY09	a	21	~693	301	150 (50%)
			b	29	~957	487	195 (40%)
				**50**	**~1,650**	**788**	**345 (44%)**
3	RNP	ssODN	a	30	~990	228	155 (68%)
			b	n.a.	n.a.	87	56 (64%)
			c	110	~3,630	200	105 (52%)
			d	11	~363	133	63 (47%)
			e	280	~9,240	182	74 (41%)
				**431**	**~14,223**	**830**	**453 (55%)**
