# Supplementary material for: Targeted mutagenesis in a human-parasitic nematode
Source: PLoS Pathog. 2017 Oct 10;13(10):e1006675. doi: 10.1371/journal.ppat.1006675 (PMC5650185; doi:10.1371/journal.ppat.1006675)
Supplement: S6 Table — Free-living adult females were injected with CRISPR-Cas9 plasmid vectors and repair template targeting either Ss-unc-22 site #2 or Ss-tax-4 site #1. Individual F1 iL3s expressing mRFPmars were genotyped for repair template integration as shown in Fig 5 and S10 Fig. (PDF) [file ppat.1006675.s016.pdf]

S6 Table. Summary of HDR in *S. stercoralis*. Free-living adult females were injected with CRISPR-Cas9 plasmid vectors and repair template targeting either *Ss-unc-22* site #2 or *Ss-tax-4* site #1. Individual F_1 iL3s expressing *mRFPmars* were genotyped for repair template integration as shown in Fig 5 and S10 Fig.

gene	target	delivery	repair	# free-living adults injected (P_0)	# red iL3s collected	# red iL3s genotyped	# repair-template-integrated iL3s	# putative -/- knockouts	% integrated
Ss-unc-22	2	DNA	pEY09	21	53	21	12	5	57%
Ss-unc-22	2	DNA	pEY09	29	55	16	12	6	75%
Ss-tax-4	1	DNA	pMLC39	21	72	19	8	0	42%
