# Supplementary material for: Targeted mutagenesis in a human-parasitic nematode
Source: PLoS Pathog. 2017 Oct 10;13(10):e1006675. doi: 10.1371/journal.ppat.1006675 (PMC5650185; doi:10.1371/journal.ppat.1006675)
Supplement: S7 Table — Summary of host passage strategies for wild-type control, 50/50 mixed unc and wild-type, and unc-enriched infections. Results for total recovery of F2 and F3 progeny for each infection strategy, and the combined nicotine-assay data presented in Fig 6B, are provided. n.a. = not available; the number of iL3s recovered was not recorded for these experiments. (PDF) [file ppat.1006675.s017.pdf]

S7 Table. Host infection and germline transmission of *Ss-unc-22* mutations. Summary of host passage strategies for wild-type control, 50/50 mixed *unc* and wild-type, and *unc*-enriched infections. Results for total recovery of F_2 and F_3 progeny for each injection strategy, and the combined nicotine-assay data presented in Fig 6B, are provided. n.a. = not available; the number of iL3s recovered was not recorded for these experiments.

I. microinjection of free-living adults, F_1 iL3 collection, and host infection

infection strategy	target	repair	# free-living adults injected (P_0)	estimated # F_1 iL3s collected	# gerbil hosts infected	estimated dose (iL3s/host)	% *unc* iL3s into host
control - wt	—	—	—	—	2	~800	0%
strategy 1 - *unc* and wt	3	ssODN	110	~3,630	2	~1,750	52.5%
strategy 2 - *unc*-enriched	3	ssODN	134	~4,422	2	~1,200	>95%

II. 1% nicotine screen of F_2 and F_3 progeny

infection strategy	life stage screened	day screened post-infection	# nematodes recovered	# nematodes screened	# twitching (%)
control - wt	F_2 or F_3 iL3s	22	~12,000	1,549	0 (0%)
		23	~14,000	1,148	0 (0%)
		24	~26,400	1,152	0 (0%)
			**~52,400**	**3,849**	**0 (0%)**
control - wt	F_2 free-living adults	25	23 (14♀, 9♂)	23	0 (0%)
		26	98 (73♀, 25♂)	98	0 (0%)
		27	178 (145♀, 33♂)	178	0 (0%)
		28	33 (19♀, 14♂)	33	0 (0%)
			**332**	**332**	**0 (0%)**
strategy 1 - *unc* and wt	F_2 or F_3 iL3s	21	n.a.	614	8 (1.3%)
		23	n.a.	700	4 (0.6%)
		25	n.a.	594	10 (1.7%)
			**n.a.**	**1,908**	**22 (1.2%)**
strategy 2 - *unc*-enriched	F_2 or F_3 iL3s	22	2,707	1,099	20 (1.8%)
		23	3,782	710	22 (3.1%)
		24	4,023	885	29 (3.3%)
			**10,512**	**2,694**	**71 (2.6%)**
strategy 2 - *unc*-enriched	F_2 free-living adults	25	21 (13♀, 8♂)	21	1 (5%)
		26	83 (59♀, 24♂)	83	6 (7%)
		27	50 (38♀, 12♂)	50	1 (2%)
		28	10 (9♀, 1♂)	10	1 (10%)
			**164**	**164**	**9 (5.5%)**
