# Supplementary material for: Targeted mutagenesis in a human-parasitic nematode
Source: PLoS Pathog. 2017 Oct 10;13(10):e1006675. doi: 10.1371/journal.ppat.1006675 (PMC5650185; doi:10.1371/journal.ppat.1006675)
Supplement: S8 Table — pPV540, provided by Dr. James Lok, was modified from pPV402, which is described in Shao et al. 2012 [45]. pEY09 and pMLC39 were modified from pAJ50, which is described in Junio et al. 2008 [31]. (PDF) [file ppat.1006675.s018.pdf]

S8 Table. Plasmid vectors for *Strongyloides* CRISPR-Cas9. pPV540, provided by Dr. James Lok, was modified from pPV402, which is described in Shao *et al.* 2012 [45]. pEY09 and pMLC39 were modified from pAJ50, which is described in Junio *et al.* 2008 [31].

construct		description	backbone
pPV540	(*Sr-eef-1A*$_{pro}$*::Cas9::Ss-era-1* 3'UTR)	*Strongyloides* codon-optimized Cas9	pPV402
pMLC56	(*Sr-U6*$_{pro}$*::Ss-unc-22-sgRNA-1::Sr-U6* 3'UTR)	sgRNA for *Ss-unc-22* site #1	pUC57-Kan
pMLC57	(*Sr-U6*$_{pro}$*::Sr-unc-22-sgRNA-1::Sr-U6* 3'UTR)	sgRNA for *Sr-unc-22* site #1	pUC57-Kan
pMLC58	(*Sr-U6*$_{pro}$*::Ss-unc-22-sgRNA-3::Sr-U6* 3'UTR)	sgRNA for *Ss-unc-22* site #3	pUC57-Kan
pMLC60	(*Sr-U6*$_{pro}$*::Ss-unc-22-sgRNA-2::Sr-U6* 3'UTR)	sgRNA for *Ss-unc-22* site #2	pUC57-Kan
pMLC61	(*Sr-U6*$_{pro}$*::Sr-unc-22-sgRNA-2::Sr-U6* 3'UTR)	sgRNA for *Sr-unc-22* site #2	pUC57-Kan
pEY09	(5'HA*::Ss-act-2*$_{pro}$*::mRFPmars::Ss-era-1* 3' UTR::3'HA)	HDR construct for *Ss-unc-22* site #2	pAJ50
pMLC47	(*Sr-U6*$_{pro}$*::Ss-tax-4-sgRNA-1::Sr-U6* 3'UTR)	sgRNA for *Ss-tax-4* site #1	pUC57-Kan
pMLC39	(5'HA*::Ss-act-2*$_{pro}$*::mRFPmars::Ss-era-1* 3' UTR::3'HA)	HDR construct for *Ss-tax-4* site #1	pAJ50
