# Supplementary material for: Targeted mutagenesis in a human-parasitic nematode
Source: PLoS Pathog. 2017 Oct 10;13(10):e1006675. doi: 10.1371/journal.ppat.1006675 (PMC5650185; doi:10.1371/journal.ppat.1006675)
Supplement: S9 Table — The PAM for each target sequence is underlined. Note that each target sequence contains guanine residues in the 1st, 19th, and 20th positions (GN(17)GG), as recommended in Farboud et al. 2015 [49]. (PDF) [file ppat.1006675.s019.pdf]

S9 Table. *Strongyloides* CRISPR target sequences. The PAM for each target sequence is underlined. Note that each target sequence contains guanine residues in the 1st, 19th, and 20th positions (GN(17)GG), as recommended in Farboud *et al.* 2015 [49].

target	sequence
Ss-unc-22 site #1	GTCCAGACCAATTCTGGTGGTGG
Ss-unc-22 site #2	GGATCAGTTGACAATAATGGTGG
Ss-unc-22 site #3	GTACAATCAGGTGTTCCAGGTGG
Sr-unc-22 site #1	GTCCAAACCAATTCTGGAGGTGG
Sr-unc-22 site #2	GGATCAACAGATAATAATGGTGG
Ss-tax-4 site #1	GTAACATTTGACTTGATGGGTGG
	**PAM**
