# Supplementary material for: Targeted mutagenesis in a human-parasitic nematode
Source: PLoS Pathog. 2017 Oct 10;13(10):e1006675. doi: 10.1371/journal.ppat.1006675 (PMC5650185; doi:10.1371/journal.ppat.1006675)
Supplement: S10 Table — Injection mixes introduced into Strongyloides free-living adult females were limited to a maximum final DNA concentration of 100 ng/μL, as previously described in Junio et al. 2008 [31]. (PDF) [file ppat.1006675.s020.pdf]

S10 Table. Plasmid vector injection mixes for *Strongyloides* CRISPR-Cas9. Injection mixes introduced into *Strongyloides* free-living adult females were limited to a maximum final DNA concentration of 100 ng/μL, as previously described in Junio *et al.* 2008 [31].

plasmid vectors *Ss-unc-22* site #1

component	concentration
pMLC56	60 ng/μL
pPV540	20 ng/μL

plasmid vectors *Ss-unc-22* site #2

component	concentration
pMLC60	60 ng/μL
pPV540	20 ng/μL

plasmid vectors *Ss-unc-22* site #3

component	concentration
pMLC58	60 ng/μL
pPV540	20 ng/μL

plasmid vectors *Sr-unc-22* site #1

component	concentration
pMLC57	60 ng/μL
pPV540	20 ng/μL

plasmid vectors *Sr-unc-22* site #2

component	concentration
pMLC61	60 ng/μL
pPV540	20 ng/μL

plasmid vectors *Ss-unc-22* site #2 + repair template

component	concentration
pMLC60	60 ng/μL
pPV540	20 ng/μL
pEY09	20 ng/μL

plasmid vectors *Ss-unc-22* site #2 + repair template (w/o Cas9)

component	concentration
pMLC60	60 ng/μL
pEY09	20 ng/μL

plasmid vectors *Ss-tax-4* site #1 + repair template

component	concentration
pMLC47	25 ng/μL
pPV540	25 ng/μL
pMLC39	30 ng/μL
