# Supplementary material for: Targeted mutagenesis in a human-parasitic nematode
Source: PLoS Pathog. 2017 Oct 10;13(10):e1006675. doi: 10.1371/journal.ppat.1006675 (PMC5650185; doi:10.1371/journal.ppat.1006675)
Supplement: S11 Table — The first 20 nucleotides of each crRNA match the genomic DNA of the CRISPR target site indicated. The remaining nucleotides are the Streptococcus pyogenes repeat sequence and are identical for all three crRNAs shown. (PDF) [file ppat.1006675.s021.pdf]

S11 Table. *Ss-unc-22* crRNA sequences. The first 20 nucleotides of each crRNA match the genomic DNA of the CRISPR target site indicated. The remaining nucleotides are the *Streptococcus pyogenes* repeat sequence and are identical for all three crRNAs shown.

target	sequence
Ss-unc-22 site #1 crRNA	GUCCAGACCAAUUCUGGUGGGUUUUAGAGCUAUGCUGUUUUG
Ss-unc-22 site #2 crRNA	GGAUCAGUUGACAAUAAUGGGUUUUAGAGCUAUGCUGUUUUG
Ss-unc-22 site #3 crRNA	GUACAAUCAGGUGUUCCAGGGUUUUAGAGCUAUGCUGUUUUG
