# Supplementary material for: Targeted mutagenesis in a human-parasitic nematode
Source: PLoS Pathog. 2017 Oct 10;13(10):e1006675. doi: 10.1371/journal.ppat.1006675 (PMC5650185; doi:10.1371/journal.ppat.1006675)
Supplement: S12 Table — RNP injection mixes were based on the injection mixes described in Paix et al. 2015 [20]. (PDF) [file ppat.1006675.s022.pdf]

S12 Table. RNP injection mixes for *Ss-unc-22* CRISPR-Cas9. RNP injection mixes were based on the injection mixes described in Paix *et al.* 2015 [20].

RNP complex for *Ss-unc-22* (w/o ssODN)

component	concentration	volume
tracrRNA	4 µg/µL	5 µL
crRNA*	4 µg/µL	0.8 µL
NF KCl	1 M	0.5 µL
NF HEPES (pH 7.5)	200 mM	0.75 µL
NF ddH_2O to 20 µL		12.95 µL

NF = nuclease-free

*crRNA *Ss-unc-22* site #1, #2, or #3

add full 20 µL of mix to 10 µg Cas9

RNP complex for *Ss-unc-22* (w/ ssODN)

component	concentration	volume
tracrRNA	4 µg/µL	5 µL
crRNA*	4 µg/µL	0.8 µL
ssODN	500 ng/µL	0.55 µL
NF KCl	1 M	0.5 µL
NF HEPES (pH 7.5)	200 mM	0.75 µL
NF ddH_2O to 20 µL		12.4 µL

NF = nuclease-free

*crRNA *Ss-unc-22* site #3

add full 20 µL of mix to 10 µg Cas9

RNP complex for *Ss-unc-22* (w/o ssODN) (w/o Cas9)

component	concentration	volume
tracrRNA	4 µg/µL	5 µL
crRNA*	4 µg/µL	0.8 µL
NF KCl	1 M	0.5 µL
NF HEPES (pH 7.5)	200 mM	0.75 µL
NF ddH_2O to 20 µL		12.95 µL

NF = nuclease-free

*crRNA *Ss-unc-22* site #2

inject without adding Cas9
