# Supplementary material for: Targeted mutagenesis in a human-parasitic nematode
Source: PLoS Pathog. 2017 Oct 10;13(10):e1006675. doi: 10.1371/journal.ppat.1006675 (PMC5650185; doi:10.1371/journal.ppat.1006675)
Supplement: S13 Table — (PDF) [file ppat.1006675.s023.pdf]

S13 Table. Primer sets used in this study.

primer set (5' → 3')		description	size (bp)	notes
forward	CCTGATATTACTGATTGGGATACTGATAGA	region around *Ss-unc-22* site #1	678	
reverse	ATAATGATCAATAGGAATTCCACCATCATC			
forward	AATTAAAATATACTTCTCTGGTTCTGCTCC	region around *Ss-unc-22* site #2	835	
reverse	TCCACCCTCATTACATGGTATAAATGTATA			
forward	CTGTCTTAGATGCACCAGGAAAAC	region around *Ss-unc-22* site #3	660	Fig 4<br>S5 Fig<br>S8 Fig - F4 x R4
reverse	ATGCAGTAATATCATGTCCAAGAGC	region for ssODN insertion + EagI digest		
forward	ATTGTTGGTGATGTTGCTGAAATTC	10 kb upstream of *Ss-unc-22* site #3	525	S5 Fig
reverse	ATAACTTGTAATTGGTGCACCTCC			
forward	ATTTTCCTGTAAAACGATTAACACCG	10 kb downstream of *Ss-unc-22* site #3	500	S5 Fig
reverse	TTTTATGCACGAATTTTGTCTTTCAATAC			
forward	TACATGGATTGCACCTGTTGATAATG	wild-type locus for *Ss-unc-22* site #2	1,092	Fig 5 - F1 x R1
reverse	ACAGGATCAGTTGACAATAATGGTG			
forward	TACATGGATTGCACCTGTTGATAATG	5' border of integration for *Ss-unc-22* site #2	1,200	Fig 5 - F1 x R2
reverse	CGAGGTACCTCTTTTCCACACTT			
forward	AAACAGAAACAGATTGGGTCTCT	3' border of integration for *Ss-unc-22* site #2	1,036	Fig 5 - F2 x R3
reverse	CATTAAATTGACCTTCAGCAGAACC			
forward	GTATTCCCTTCTATTGTTGGAAGACC	genomic DNA control (*Ss-act-2* exon 1)	416	Fig 4<br>Fig 5<br>S5 Fig
reverse	CCTTCATAGATTGGTACAGTGTGAG			
forward	ATTAAAGAGGCATGGGATCTTGATG	5' homology arm for *Ss-unc-22* site #2	640	
reverse	GACCTTGTCCATTTTCATTTACTGC			
forward	TAGCAACTCTTCCATTTAATCCACC	3' homology arm for *Ss-unc-22* site #2	633	
reverse	ATTTAGGTGGTACAAAAACACGTGG			
forward	TGACAAAAATTCAAAATGCTCCTGG	wild-type locus for *Ss-unc-22* site #3	960	S8 Fig - F3 x R4
reverse	ATGCAGTAATATCATGTCCAAGAGC			
forward	TAATACGACTCACTATAGGG (T7)	ssODN integration for *Ss-unc-22* site #3	356	S8 Fig - T7 x R4
reverse	ATGCAGTAATATCATGTCCAAGAGC			
forward	TTATCCACCCCGAAGAAGTAAGC	wild-type locus for *Ss-tax-4* site #1	1,822	S10 Fig - F5 x R5
reverse	CCCAACAGATCTAACATTAGCCG			
forward	TTATCCACCCCGAAGAAGTAAGC	5' border of integration for *Ss-tax-4* site #1	1,350	S10 Fig - F5 x R2
reverse	CGAGGTACCTCTTTTCCACACTT			
forward	TGATCACGACTCTGAGTTGACTG	5' homology arm for *Ss-tax-4* site #1	1,001	
reverse	CTGGACCAGGAACTTCACCAATA			
forward	CGTCTAACGGCTGAATATACTAA	3' homology arm for *Ss-tax-4* site #1	1,045	
reverse	TGTTTTCGCTTCTTTTGATGACA			
