# Supplementary material for: Targeted mutagenesis in a human-parasitic nematode
Source: PLoS Pathog. 2017 Oct 10;13(10):e1006675. doi: 10.1371/journal.ppat.1006675 (PMC5650185; doi:10.1371/journal.ppat.1006675)
Supplement: S14 Table — Injected free-living adult females were reared on host feces based on the observation that it results in higher reproductive output relative to other standard culturing methods [53]. iL3s were collected using a Baermann apparatus and all F1 progeny were counted to calculate the average number of iL3s per injected adult. (PDF) [file ppat.1006675.s024.pdf]

S14 Table. The average number of F_1 iL3s collected per microinjected free-living adult female for *S. stercoralis* and *S. ratti*. Injected free-living adult females were reared on host feces based on the observation that it results in higher reproductive output relative to other standard culturing methods [53]. iL3s were collected using a Baermann apparatus and all F_1 progeny were counted to calculate the average number of iL3s per injected adult.

species	post-injection culturing method	# free-living adults injected (P_0)	# F_1 iL3s collected	# F_1 iL3s / injected adult
S. stercoralis	fecal charcoal	29	862	29.7
	fecal charcoal	30	909	30.3
	fecal charcoal	29	1,113	38.4
	fecal charcoal	37	1,105	29.9
	fecal charcoal	32	1,196	37.4
				**33 iL3s / injected adult**
S. ratti	chemotaxis plate + rat feces	45	1,012	22.5
	fecal charcoal	20	358	17.9
	fecal charcoal	40	1,319	33.0
				**25 iL3s / injected adult**
